# In silico exploration of mouse brain dynamics by focal stimulation reflects the organization of functional networks and sensory processing

**DOI:** 10.1162/netn_a_00152

**Published:** 2020-09-01

**Authors:** Andreas Spiegler, Javad Karimi Abadchi, Majid Mohajerani, Viktor K. Jirsa

**Affiliations:** Department of Neurophysiology and Pathophysiology, University Medical Center Hamburg-Eppendorf, Hamburg, Germany; Canadian Center for Behavioural Neuroscience, University of Lethbridge, Alberta, Canada; Canadian Center for Behavioural Neuroscience, University of Lethbridge, Alberta, Canada; Institut de Neurosciences des Systèmes, UMR Inserm 1106, Aix-Marseille Université, Faculté de Médecine, Marseille, France

**Keywords:** Large-scale network, Brain modeling, Stimulation, Sensory stimuli, Voltage-sensitive dye, Brain dynamics, Connectivity, Resting state

## Abstract

Resting-state functional networks such as the default mode network (DMN) dominate spontaneous brain dynamics. To date, the mechanisms linking brain structure and brain dynamics and functions in cognition, perception, and action remain unknown, mainly due to the uncontrolled and erratic nature of the resting state. Here we used a stimulation paradigm to probe the brain’s resting behavior, providing insights on state-space stability and multiplicity of network trajectories after stimulation. We performed explorations on a mouse model to map spatiotemporal brain dynamics as a function of the stimulation site. We demonstrated the emergence of known functional networks in brain responses. Several responses heavily relied on the DMN and were suggestive of the DMN playing a mechanistic role between functional networks. We probed the simulated brain responses to the stimulation of regions along the information processing chains of sensory systems from periphery up to primary sensory cortices. Moreover, we compared simulated dynamics against in vivo brain responses to optogenetic stimulation. Our results underwrite the importance of anatomical connectivity in the functional organization of brain networks and demonstrate how functionally differentiated information processing chains arise from the same system.

## Author Summary

We demonstrate how functionally differentiated information processing chains arise from the same anatomical network. The main result of the in-silico mouse brain simulations is the emergence of specific functional networks based on structural data from the mouse brain. When the brain is stimulated, for example, by sensory inputs or direct electrical stimulation, the brain initially responds with activities in specific regions. The brain’s anatomical connectivity constrains the subsequent pattern formation. We built a high-resolution mouse brain network model. The model structure originated from experimental data. We systematically explored the mouse model and investigated the simulated brain dynamics after stimulation. Known functional networks emerged in the simulated brain responses. The default mode network occurred in almost all characteristic response patterns. Simulated brain response dynamics and in-vivo response dynamics of the mouse brain to optogenetic stimulation showed similarities even without parameter tuning. Anatomical connectivity and dynamics shape the functional organization of brain networks.

## INTRODUCTION

Many structural and functional components of brain networks are known across spatial and temporal scales. However, a computational integration framework enabling us to link brain activity to its function in cognition, perception, and action is missing. The debate on the neural representation of behavior is longstanding and ongoing (see recent discussions, Huys et al., 2014; Krakauer et al., 2017; Pillai & Jirsa, 2017) and is currently converging toward acknowledging its dynamic nature. Mathematical models such as Structured Flows on Manifold (see Pillai & Jirsa, 2017), Dynamical Causal Modeling (DCM; Friston et al., 2003), and Heteroclinic Cycles (Rabinovich et al., 2008) are used to capture and model dynamical brain behaviors. However, when it comes to large-scale brain activity, current models remain dominated by static representations of brain activity, that is, mapping of brain states including sleep, seizure, and behavior (e.g., sniffing). Such large-scale brain studies are prone to neo-phrenological interpretations and localizationist perspectives.

An example is the representation of functions in cognition, perception, and action in brain activity, observable in the magneto- (MEG) and electroencephalogram (EEG), as well as in functional MRI (see, for instance, Dehaene et al., 2015; Kringelbach et al., 2015; Fuchs et al., 2000; Calvert et al., 2004, and the discussion therein). To go beyond the sampling of brain states and localization of their activity focus, brain activity/dynamic outside its attractor states needs to be systematically measured and evaluated from a dynamics perspective (Deco et al., 2011, 2012, 2013). The spontaneous brain activity at rest, that is, in the absence of any specific task, defines one of such nonattractor brain states. Spontaneous and task-related activity exhibits correlated activity patterns, called resting-state functional networks (e.g., Mantini et al., 2007). Such networks have been identified by investigating resting- and task-related brain dynamics and sequences of brain activation patterns (microstates: Van De Ville et al., 2010; Michel & Koenig, 2018), fitting low-dimensional dynamical systems to event-related potentials (for instance, in generative Bayesian models via DCM: David et al., 2006; Ryali et al., 2016), and deriving estimators of various forms of causal interactions among brain areas (Wang et al., 2008). Such inversion analysis relies on all cases on the model in use, the inversion data features, and the model’s identifiability (Raue et al., 2009). However, in silico brain modeling avoids the issues associated with model inversion and allows creating brain network dynamics on top of biologically realistic structural connectivity (SC). When such brain models are implemented in an appropriate neuroinformatics framework (see, for instance, http://www.thevirtualbrain.org), biophysical forward solutions, linking the brain source signals to functional neuroimaging signals (e.g., M/EEG, BOLD, and MRI) can be computed. In silico modeling of stimulated brain activity thus offers an excellent means of exploring brain dynamics inside and outside of attractor states, with a direct link to measurable brain signals.

**Figure 1.**
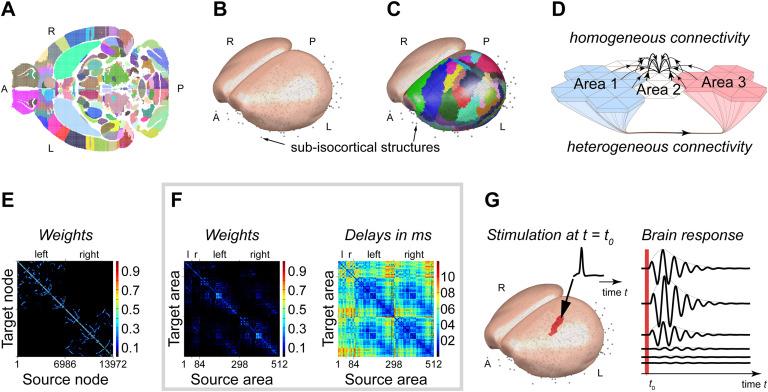
Modeling the mouse brain and simulating the mouse brain’s response to focal stimulation. The ABA database is isotropically sampled with a resolution of 50 *μ*m, resulting in a total of 3,300,706 voxels (1,653,785 voxels for left side), which encode the injection by its spatial location and connectivity among 512 brain areas. Supporting Information Tables S1 and S2 list the 512 brain areas. (A) An axial (horizontal) slice of the sampled database. Each dot indicates the center of a voxel, and the color indicates the different brain areas. To model large-scale activity of the cortex recorded using various methods such as VSD imaging, the isocortex surface is reconstructed from the ABA database (slice shown in panel A) using a regular triangle mesh of 7,020 vertices for the left and 6,952 vertices for the right hemisphere. The quality of the mesh is quantified in Supporting Information Figure S2 shows the quality of the mesh. (B) The reconstructed isocortical surfaces. All other areas are lumped in space to point masses (centroids) indicated by the gray circles in panels B, C, and G. (C) Each vertex in the isocortical meshes is assigned to an area by the five closest voxels to the normal vector extended inside the volume of the ABA (slice shown in panel A). Table 1 lists the areas of the isocortex and the number of vertices per area. (D) The model distinguished two types of structural connectivity (SC). Long-range SC corresponds to the connections extracted from the ABA, such as tracts connecting brain areas over long distances. Short-range SC corresponds to gray matter fibers in the isocortex, with short-range connections within a given isocortical area. It also enables some communication over short distances between neighboring areas. Although in the ABA, cortical area 2 is not connected to areas 1 and 3, it is weakly linked to both areas via a set of short-range SC. (E) Short-range SC matrix for the 13,972 vertices of the isocortex. The synaptic weights are color coded. The diagonal describes in warm colors the strong SC of adjacent nodes. Cold colors show the SC decreases with distance. SC of nearby nodes are scattered (e.g., blue dots) in panel E because each cerebral hemisphere is described by a surface, which makes it impossible to cluster nodes locally along both vertical and horizontal axes of the weight matrix. Note the absence of interhemispheric short-range SC. (F) Long-range SC for the 512 (84 isocortical plus 428 subcortical) brain areas for weights (left) and time delays (right). Within one hemisphere, the 214 subisocortical areas mostly project to the 42 isocortical areas. Some connections between subcortical areas can also be seen. The 42 cortical areas project heavily to both cortical and subcortical areas. Some interhemispheric connections can also be seen. Note also the presence of relatively large time delays. (G) Stimulation paradigm. Dynamics are simulated without noise. Brief stimulation induces activity in the targeted brain area. Panel G shows the mouse brain model responding (at different locations) to stimulation of the primary somatosensory area. ss-DRN captures the spreading and dissipating activity via the SC. Each of the 512 cortical and subcortical brain areas is stimulated individually, and the ss-DRNs are extracted and investigated.

To perform systematic exploration of the brain dynamics via stimulation in silico, we constructed a large-scale network model with homogeneous short-range SC, the axonal connection between brain areas, and region-specific long-range SC in the isocortex (Figure 1). In our description, boundary-crossing is possible. That is, short-range fibers can project to not only the region from which they emanate but also to adjacent regions. To eliminate the impact of diverse local activity on whole-brain dynamics, we used the same local dynamics (a simple oscillator with the same parameters; see Supporting Information Figure S1) to represent the activity of each network node. It allowed us to isolate the role of network anatomical structure on whole-brain dynamics. Using the same local dynamics, we probed the experimentally derived large-scale brain network to produce functionally relevant activity using focal stimulation. The stimulus-related activity can spread from the stimulation site and synchronize in different locations on the network over time.

To investigate whole-brain dynamics, we performed simulations and analyzed brain responses to brief stimulation. Stimulation of a brain area induced spatiotemporal patterns of activity dissipating energy over the network. We systematically stimulated brain areas and compiled a catalog of brain responses. Individual trajectories in the responses provided fingerprints of the mouse brain. The main result of in silico modeling is the appearance of specific functional networks in the simulated brain responses.

To determine the dominant activity pattern of coactivated brain regions, we decomposed each brain response. Three principal components (PCs) defined the stimulation site-specific dynamically responsive network (ss-DRN), in which 12 consistent dynamically responsive networks (DRNs) appeared across stimulation sites. The induced activity resolved in one of the DRNs, irrespective of the stimulation site. The 12 DRNs had five recurring activity patterns. These motifs correlate with functional networks (Sforazzini et al., 2014; Zingg et al., 2014). The salience network and the default mode network (two well-characterized brain functional networks) occurred in almost all DRNs, indicating a connecting role for both networks between the functional networks.

Using our novel network model of the mouse brain that permits systematic investigation of peripheral pathways, especially the ones for sensory data processing, we showed that the simulated in silico brain responses resembled in vivo brain dynamics after stimulation. Interestingly, the simulated data bore a noticeable similarity to the empirical data, collected using voltage-sensitive dye (VSD) imaging and laser stimulation in mice, even though we had not attempted to fit our model to this empirical data.

## RESULTS

The result section splits into two parts: (1) compiling of the whole mouse model and (2) the in silico exploration study utilizing focal stimulation. The modeling work is fundamental to the in silico exploration and is also suitable for many studies, and in this sense, the modeling work stands for itself. The modeling work of the mouse brain was thus not solely driven by the goal of performing in silico brain stimulation but is instead driven by the objective of obtaining a systemic and holistic description of the mouse brain. The results emphasize the potential of dynamic models to study the functional repertoire of the brain and the information processing of networks.

### Modeling the Mouse Brain

The Virtual Brain (TVB) network modeling approach involves three interacting systems: the inputs, the dynamics, and the observations. The *input system* allows for interventions, the *observer system* specifies the measurement modalities, and the *dynamical system* generates the brain activity based on its *structure* and constitutes brain states. The physical and spatiotemporal properties of actions (e.g., focal brief, direct electrical stimulation, or transcranial direct current stimulation) to brains determine the input to TVB. In general, models are agnostic about the action. They hence have the potential to integrate and discuss the effects of different modalities such as sensory, direct electrical, and transcranial stimulation as well as blocked arteries. The input system relates action to the state variables of TVB (e.g., postsynaptic potentials and currents). The dynamical system describes the interactions between state variables and their spatiotemporal evolution. As a result, the dynamical system produces a set of events (over a relatively short timescale, e.g., event-related potentials (e.g., Shah et al., 2004) and event-related (de)synchronization (Pfurtscheller & Lopes da Silva, 1999), and long-term effects such as necrosis and changes in apoptosis and autophagy). Such events may be symptomatic for medical conditions and diseases such as ischemic stroke (a blocked artery) and multiple sclerosis (due to damage of myelin sheath). In the following, we present the modeling results for the mouse brain and the VSD imaging data in detail.

Structure and parcellation of the mouse brain model originated from the Allen Brain Atlas (ABA; Oh et al., 2014). It compiles the axonal projections labeled by viral vector tracer injections to a selective sample of adult mice brain regions (see Figure 1). The sampling is with high spatial resolution such that the model distinguishes 512 distinct brain areas. From the ABA, we reconstructed and approximated the smooth surface of the isocortex by using a regular mesh (Supporting Information Figure S2). We then spatially divided the isocortex into cortical areas given by the ABA. The 512 distinct brain areas comprise 84 isocortical areas (i.e., 42 per hemisphere) and 428 nonisocortical (subcortical). Table 1 lists the division of structures into areas. Supporting Information Table S1 and S2 provide a complete list of area names. Short- and long-range SCs connected brain areas. Both SCs are exclusively excitatory. Inhibition, however, is implicitly present in the local dynamic models to dissipate activity.

**Table 1.** Mapping of brain areas in the mouse model

Structure	Division	Areas
Cortex	Isocortex	42
	Olfactory areas	11
	Hippocampal formation	10
	Cortical subplate	7

Cerebral nuclei	Striatum	13
	Pallidum	8

Thalamus	Sensorimotor related	13
	Polymodal association cortex related	8

Hypothalamus	Periventricular zone	3
	Periventricular region	11
	Medial zone	12
	Lateral zone	7

Midbrain	Sensory related	6
	Motor related	17
	Behavior-state related	7

Pons	Sensory related	3
	Motor related	7
	Behavior-state related	7

Medulla	Sensory related	9
	Motor related	23

Cerebellum	Cortex	12
	Nuclei	3


*Note*. Mapping of brain areas in the mouse model refers to areas of brain structures following the division of the Allen Brain Atlas (http://connectivity.brain-map.org). Because of a total of 512 brain areas in the model, only the number of areas is listed. Names are listed in Supporting Information Table S1 and S2.

Short-range SC comprises axonal projections within the isocortex (e.g., in a brain region) over distances of micrometers up to millimeters (Braitenberg & Schüz, 1998). Anisotropic radial kernel described these short-range connections in our model for which the SC decays as a function of distance for the center (Braitenberg & Schüz, 1991). Also, the same kernel was applied homogeneously throughout the entire isocortex. Short-range SCs described by these kernels allowed crossing the boundaries of brain areas. The model implemented short-range SCs only in the isocortex and not for the rest of the brain.

Long-range SC comprises axonal connections between distant brain areas. The ABA provides these connections between the 512 distinct brain areas of the adult mouse brains. These connections are directional (e.g., the SC of lateral geniculate complex (LGN) to V1 is stronger than that in the other direction)), which introduces asymmetry and specificity for connectivity between a pair of brain areas leading to the heterogeneity of long-range SC. Adjacent brain areas in the isocortex exchange information via the short-range SC and, if available (by ABA), via the long-range SC. At each network location, the activity enters and exits via afferent and efferent projections whose weights are experimentally derived. These weights indicate how strong a location in the network model collects and distributes activity—in other words, how well the brain network embedded brain structures. It turned out that the hypothalamus is the most embedded brain structure in the mouse brain model based on the ABA (Supporting Information Figure S3 and S4). The hypothalamus and the cerebellum receive the strongest and weakest input in the mouse brain model, respectively. Despite ample connections, the graph-theoretic measures point, on average, to weakly connected isocortex with the subcortex (see Supporting Information Figure S3). Hence, the dynamics of isocortex can exert functions that are less sensitive to subcortical input from via long-range SC.

Tracer injection techniques used for the ABA captures unidirectional connectivity patterns. The ABA-derived long-range SC weight matrix was highly symmetric, pointing to a dominant bidirectionality of the long-range SC. The used measures (described in Materials and Methods) indicated high symmetry in the long-range SC by low values *Q*_0_ = 238.2871 × 10^−3^ and *Q*_1_ = 226.6417 × 10^−3^ (*Q*_0_ and *Q*_1_ range between 0 and 1; 0 for perfect symmetry and 1 for asymmetry and antisymmetry in *Q*_0_ and *Q*_1_, respectively).

The local dynamic model allows each location in the mouse brain network to show rhythmic activity in the gamma range (at about 42 Hz), accounting for the coordinated interaction of excitation and inhibition in local circuits (Buzsáki & Wang, 2012; Palmigiano et al., 2017) that has been phenomenologically modeled by Palmigiano et al. (2017). A two-dimensional (2D) model (i.e., consisting of two state variables: mean postsynaptic potential and current) was used to provide the local temporal dynamics (see, e.g., Spiegler et al., 2016). We parameterized this 2D local dynamic model equally across the locations of the mouse brain network model. That means, each isolated location in the brain network (with SC removed) behaves identically. Note that the behavior at each network location was set to a subcritical working point, as many authors suggest within the resting-state literature (Deco et al., 2011; Fagerholm et al., 2015). A stable focus close to instability is here subcritical by showing damped oscillations in response to perturbations.

In contrast to many other modeling studies, we considered the deterministic system only to investigate brain responses to stimulation. It did not include dynamic noise to explore the state space for describing ongoing brain activity. Hence, without stimulation or perturbation (no noise, no nonzero time-varying input), activity was zero. Local response to stimulation was a damped oscillation in the gamma frequency range. The long-range SC of the mouse brain was heterogeneous (see Supporting Information Figure S5). The spatiotemporal patterns of activity, generated by the model, were shaped by the brain structure through the network interactions. Hence, the local response can be highly specific to a brain area even though local dynamics were identical. The specific connections to a given location in the mouse brain network and the transferred activity from a source location pushes the operating point of the target location toward the critical point (i.e., Andronov–Hopf bifurcation) at which the decay time of a stimulation response increases. We parameterized the brain model in a way that none of the applied stimulation can lead to a self-sustained oscillation. Consequently, the brain network segregates and integrates information, while the local dynamics dissipate the activity.

Transmission speed and delays originated from mouse connectivity and physiological studies. Following the reasoning of (Buzsáki & Wang, 2012), the activity of local neural populations in the brain shows gamma oscillations (30–90 Hz) with cycle duration between 11 ms and 33 ms. Let us consider the axonal lengths (20 mm to 40 mm) between lateral areas of two hemispheres in mice (Schüz et al., 2006). The effective conduction speed should be less than 3.6 m/s (for the extreme with the longest axon (40 mm) and the fastest local activity (11 ms), assuming that the timescales of transmission delays have to be similar to the characteristic timescales of local brain activity. This estimation is in line with the range of conduction speed found in myelinated visual cortical neurons whose axons pass through the corpus callosum in mice (Waxman, 2012). Most of the cortico-cortical homolateral connections in mice are shorter than 10 mm. For instance, Braitenberg and Schüz (1998, Chapter 26) report the longest distance of 7 mm to the tracer injection site in a planar view on the isocortices with two modes, at about 2 mm and about 3.5 mm. The effective conduction speed is estimated to be less than 1 m/s for a maximum connection of 11 mm divided by 11 ms to affect fast (90 Hz) gamma oscillation. The upper bound of the speed for the longest distance to the tracer injection site and the two modes of distance to the tracer injection site (see Braitenberg & Schüz, 1998, Chapter 26) are 0.6364 m/s = 7 mm / 11 ms, 0.1818 m/s = 2 mm / 11 ms, and 0.31818 m/s = 3.5 mm / 11 ms. Transmission delays were assumed to affect intrinsic local activity hardly, that is, gamma waves, which are most apparent at a frequency of >40 Hz across the brain. With a modest speed of 3 m/s and a 10 mm connection length, the time delay is up to 3.333 ms. The delay is rather short and plays a limited role in the mouse brain, especially if the speed can be slowed down by a factor of 2 to 3. Thus, transmission delays could hardly affect intrinsic local activity, that is, gamma waves, which are the most apparent at a frequency of >40 Hz across the brain.

The time delay can be effective on a scale smaller than the characteristic time constant of the intrinsic frequency (i.e., the period of a cycle), considering harmonic oscillations (i.e., sinusoidal activity) in the brain. Moreover, we expect time delays to be most effective within a quarter of a full cycle because of the change in the wave. It results in effective time delays of around 6.25 ms for 40 Hz and 2.77 ms for 90 Hz. Therefore, short and long time delays affect mainly fast and slow oscillations, respectively. Since the majority of connections are about 6.25 mm long (and 2.77 mm for 90 Hz), we equipped each location in the mouse brain network with simple dynamics. Each location responds with a damped oscillation in the gamma range (at about 42 Hz) to stimulation and incoming input from other brain regions (see Supporting Information Figure S1). The chosen intrinsic frequency for local dynamics leads to the effective conduction speed of about 1 m/s.

The *conduction speed* for white matter fiber tracts was assumed in the model to be 1 m/s. Given the distances between the brain areas present in the ABA, the transmission delays result from the division of the mean connection lengths by the conduction speed (see Supporting Information Figure S5). First of all, the histograms in Supporting Information Figure S5 do not indicate for bimodality, as reported by Braitenberg and Schüz (1998, in Figure 62, Chapter 26). However, the technique is similar, and the distance measurement is slightly different. Our investigation of intra- and interhemispheric SC, however, confirmed the second peak in the histogram (see Supporting Information Figure S5B). The investigation is based on the division of the mouse brain into areas and intra- and interhemispheric connections (e.g., among left isocortical areas and between left and right isocortical areas, respectively). This second peak reflects the long commissure connecting the two hemispheres, such as the corpus callosum. The two peaks in the histogram for intra- and interhemispheric connection lengths are not visible in the histogram of all isocortical connection lengths. The reason is that the number of connections is similar within and between hemispheres of the isocortex (Table 2). The same is true for all the connections in the entire mouse brain model (see Supporting Information Figure S5A). Table 3 summarizes the descriptive statistics of the connection lengths.

**Table 2.** Number of connections among areas in the isocortex is outnumbered by other areas in the mouse brain model

Area	Structure		
	Mouse brain (512)	Isocortex (84)	Subisocortex (428)
Intra	127,922 (97.98%)	003,444 (100%)	088,820 (97.43%)
Inter	127,814 (97.51%)	003,528 (100%)	088,788 (96.94%)
All	255,736 (97.75%)	006,972 (100%)	177,608 (97.18%)

*Note*. The table lists the number of connections of the mouse brain model and its division into isocortex and subisocortex. In brackets are the numbers of different areas or the percentages of connections concerning possible connections.

**Table 3.** Connection lengths in the mouse brain model and its subdivisions

Parameter	Mouse brain		Isocortex		Subisocortex	
	Intra	Inter	Intra	Inter	Intra	Inter
Minimum	0.1085	0.1083	0.4441	0.2352	0.1085	0.1083
Maximum	11.6731	11.8715	8.5598	10.0594	11.6731	11.8715
Expectation	4.0562	4.9721	3.7661	6.3171	3.6806	4.4273
Variance	3.3677	3.4961	2.8349	3.5418	3.0860	2.9773
Skewness	0.5133	0.2307	0.2042	−0.5012	0.6632	0.3889
Quantile-05%	1.3444	2.0139	1.1605	2.7377	1.1909	1.7948
Quantile-50%	3.8858	4.9148	3.6685	6.5294	3.4521	4.3090
Quantile-95%	7.2747	8.1711	6.5422	9.0240	6.8353	7.3476
Quantile-99%	9.0197	9.4866	7.7075	9.5383	8.4382	8.7810

*Note*. Note the difference between intra- and interiscocortical connections in terms of expectation, median (i.e., 50% quantile), and variance. All values are in millimeters, except the variance that is in square millimeters and the skewness that is a bare number.

### In Silico Exploration of Mouse Brain Dynamics by Means of Stimulation

In in silico exploration, we have varied three parameters, namely, the following:

1.  The scaling factor of the SCs to control the effectiveness of short- and long-range SCs (only long-range SC for *α* = 0, only short-range SC for *α* = 1, a mix of both SCs for 0 < *α* < 1)
2.  The characteristic distance of short-range SC within plausible bounds (Braitenberg & Schüz, 1998)
3.  The location of the stimulation according to the brain areas provided by the ABA

### Effect of Structural Connectivity with Time Delays on the Brain Response to Stimulation

The formation of consistent patterns of coactivated brain regions due to stimulation, that is, the ss-DRNs, was different for extreme sides of the scaling of SC (i.e., 0% (100%) and 100% (0%) of long-range (short-range) SC; see Supporting Information Figure S6). The occurring ss-DRNs differ at both extremes because of the change in communication via short-range and long-range SCs. The results indicate that both extremes merge rather than reorganize (e.g., as an effect of time delays in the long-range SC) as a function of each connectivity type. One explanation for this behavior is that the short-range SC pertains to a minority of areas present in the model, that is, 84 isocortical out of 512 areas. In contrast, the long-range SC (which is heterogeneous and brain area specific) pertains to all of the 512 areas. The ss-DRNs were most similar when the 512 areas were linked only by long-range SCs. The overall similarity decreases as the proportion of short-range (long-range) SC increases (decreases), and the differentiation of clusters of similar ss-DRNs becomes clearer (see Supporting Information Figure S6). The cluster labels of ss-DRNs, however, stayed the same throughout. Structures within a cluster may appear for higher scaling of the short-range SC, but clusters do not move, split, nor merge (as observed in a similar human study; Spiegler et al., 2016). The behavior in the similarity of ss-DRNs moderately depended on the spatial range of the short-range SC only for higher scaling of short-range SC (e.g., 80%). All in all, both SC types of short- and long-range SCs in the mouse model act as spatiotemporal filters of local dynamics in the emergent functional network. The results (Supporting Information Figure S6) do not provide evidence for a reorganization of dynamics due to interplay between both types of SC in the mouse as opposed to human models (Spiegler et al., 2016).

### Dynamically Responsive Networks

**Figure 2.**
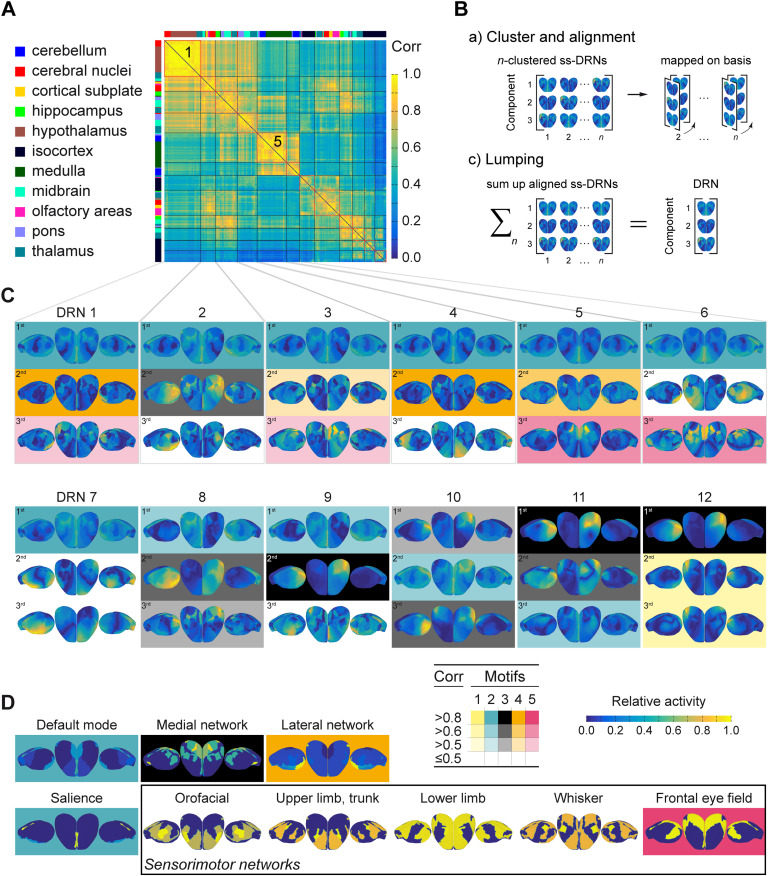
Motifs in the DRNs extracted from the brain responses in the mouse brain model. (A) The similarity matrix of the 512 ss-DRNs. Clustering the stimulation sites according to the correlation of ss-DRNs revealed 12 clusters (separated by the black lines). This suggests that similar networks are activated in response to the stimulation of several distinct areas. The color bar on the *x*-axis and the *y*-axis displays the structures to which a brain area belongs. The similarity matrix of ss-DRNs is color coded (warmer color means more similarity). The three principal components of each ss-DRN cover more than 99% of the activity variance. (B) The correlated components (patterns) of clustered ss-DRNs were aligned with the ss-DRN that shows the strongest correlation and is averaged to a characteristic DRN. (C) The 12 DRNs accessible from different locations in the brain (see panel **A**). Panel C displays the spatial activity (topography) of each of the three components that form a DRN from the left, top, and right view of the isocortex. Similar components among the DRNs are shaded in panel C (yellow, teal, gray, orange, magenta) and show five motifs that recur in the DRNs. Experimentally known functional networks were compared to the components. Shade indicates the correlation level (Corr) of the components (no shade, i.e., white for not significant correlations and shades: dark, medium, light for significant correlation values greater than 0.8, 0.6, 0.5). Six components are unique (i.e., in DRNs 2, 4, 6, 7, and 9) and not correlated to other DRNs. That makes DRN 7 the most unique one, and DRN 1 the most entangled motif (followed by DRNs 5, 11, and 12). (D) The organization of brain activity in the isocortex for the nine functional networks from the left, top, and right view (blue to yellow scale gives the relative contribution). Four experimentally known functional networks match the motifs. They are shaded, according to panel **C**. The (lateralized organization) of the frontal eye field network is the only sensorimotor network that is reflected in the DRNs (magenta motif 4). The white components in panel **C** (DRNs 2, 4, 6, 7, and 9) are unique and do not match any of the functional networks. Similarly, no match was found for motif 1 (yellow), but the pattern is not unique and recurs. The default mode and the salience networks (teal motif 2) cover most of the DRNs (1 to 11), emphasizing their sensitivity and flexibility. In contrast, the lateral (orange motif 4 in DRNs 1, and 3 to 5) and the medial network (gray motif 3 in DRNs 8 to 12) are less accessible, where the DRNs reflect the lateral organization of both networks. The model configuration is 500-μm short-range SC with 40% of short-range SC to 60% of long-range SC.

The spatial proximity of stimulation sites did not necessarily predict the similarity of induced activities. The stimulation of several sites scattered all over the brain can induce the same spatial pattern of brain activity (Figure 2). So there should be stable spatial patterns that underlie similar ss-DRNs. Such stable patterns are called the DRN in contrast to the stimulation ss-DRNs. The following three steps determined the DRNs: (1) each ss-DRN was decomposed into three PCs conveying 99% of the stimulation-induced energy (i.e., integral of power over time), (2) ss-DRNs were clustered into 12 clusters (12 was the optimum number; see Materials and Methods) based on the similarity of their corresponding PCs, and (3) the PCs of all the ss-DRNs in a given cluster were aligned, and then averaged. The resultant three averaged PCs in a given cluster defined the components of the DRN associated with that cluster. DRNs were consistent in response to different stimulation sites and consistent throughout the systematic connectivity parameter exploration (Figure 2 and Supporting Information Figure S6). Therefore, in silico exploration provided a spatiotemporal mapping of brain activity onto DRNs, which inform what brain areas to stimulate (Figure 2A: the similarity matrix) to “push” the brain into a particular state (see the catalog in Figure 2C and 2D). Please note that for the rest of the manuscript, we refer to the 12 DRNs without indicating the stimulation side (left/right) because of bilateral symmetry (in the spatial organization of brain activity due to left/right stimulation).

### Disentangle the DRNs from the Structure

We have performed statistics on the catalog of DRNs (Figure 2) to (1) clarify whether the DRNs are entangled in their components, and (2) to discuss how the formation of the stimulus-induced brain activity follow the structure. For the first purpose, the analysis revealed five significantly distinct spatial patterns in the DRNs, indicating that some of the DRNs are entangled. Because these patterns recur in several DRNs, we call them motifs. Distinct motifs could represent structural links for the transfer of brain activity at rest, transmitting information about stimuli from one DRN to another. The reason for that conjecture is the occurrence of five motifs in 12 DRNs (see the highlighted components in Figure 2), which, importantly, also allow activation of more than one DRN at a time. The analysis also revealed six unique components (i.e., see the components with white background in the DNRs 2, 4, 6, 7, and 9 in Figure 2A) that were not correlated to other DRNs. Considering the components of the DRNs and the occurrence of motifs, DRN with number 7 (see Figure 2A) is the most unique one, and DRN with number 1 is the most entangled one (followed by the entanglements in the DRNs 5, 11, and 12) among all DRNs. The motif with number 2 (shaded in teal) is the most present in the DRNs (part of 11 DRNs out of 12), whereas motif 1 occurs only within DRN 12. The most present motif, that is, number 2 (shaded in teal), was also the pattern with the strongest correlations (out of a total of 11 components, seven components were strongly correlated, and four components showed good correlation), whereas motif 1 showed only good correlations. Before interpreting the theme of the DRNs, we focused on relating the components of the DRNs (and thus the motifs) to experimentally known functional networks and tested to which extent the topology in the SC predicts the brain activity induced by stimulation.

The brain activity in response to stimulation follows the structural connections from the stimulation sites. Among the 13 graph-theoretic measures on the long-range SC, the direct projections from the stimulation sites were the best predictor for brain response, though the correlation was rather moderate. The other 12 graph-theoretic measures (see Materials and Methods) were poor, that is, less correlated, weakly predictive, and with fewer significant configurations. The best prediction resulted from comparing the rank (Kendall’s tau) of the activity at network locations after stimulation with the rank of connectivity strengths of the direct projection from the stimulation site. The rank correlation was weak but significant for most SC parameter configurations, that is, scaling *α* of long- and short-range SC and the spatial range of short-range SC. By utilizing this simple measure on the large-scale SC, a high correlation value means that energy (i.e., integral of power over time) induced by the stimulation spreads into brain areas directly connected to the stimulation site. The values of (Bonferroni corrected) significant correlations were, however, reasonably low with an average maximum value of 221.542 × 10^−3^ across all components of all DRNs (i.e., isocortex) and all connectivity parameter configurations (maximum average correlation value of 253.889 × 10^−3^ for all first components, 255.936 × 10^−3^ for all second components, and 338.724 × 10^−3^ for all third components across all DRNs and all SC parameter configurations). The percentage of parameter configurations with significant rank correlation is 48.981% across all components and all DRNs (49.706%, 56%, and 82.576% for the first, second, third components, respectively). This low rate of significant configurations and the overall low correlation indicate that most of the activity induced by the stimulation spreads over the network on short and long ranges in specific ways. The “direct network response” to stimulation seems to be captured by the second and third components of the DRNs because correlations were generally more reliable for higher order components, that is, second and third components than for the first components (and across all DRNs). The first components of each DRN reflect the reorganization of the network activity caused by the stimulation. Because of the reorganization, these spatial formations of brain activity were not necessarily traceable to the site at which stimulation introduced energy.

When considering stimulation-induced activities in the entire brain model (i.e., including all substructure), the maximum correlation was the lowest. However, the number of parameter configurations with significant correlations was the highest, which emphasizes the specificity of responses of the brain (e.g., thalamus, isocortex). For instance, the cerebellar activity showed the highest maximum correlation with the direct projections from the stimulation sites. In contrast, the number of configurations with a significant correlation was reasonably low. It is also interesting to mention that activity in the isocortical subplate showed no significant correlation with the underlying structure.

### Comparison of Dynamically Responsive and Functional Networks

To probe the potential relationship between the identified DRNs and brain function, we compared the DRNs with previously identified functional networks. Spatial organization of the nine experimentally known functional networks derived from Sforazzini et al. (2014) and Zingg et al. (2014) is shown in Figure 2B for the isocortex. Four of the nine functional networks matched four of the five motifs in the DRNs (see Figure 2). Interestingly, the functional sensorimotor networks were not similar to any of the DRNs. Merely the lateral organization (i.e., activity within one hemisphere) of the network associated with the frontal eye field was significantly (but moderately) reflected in the magenta motif five that links four DRNs (see Figure 2). A reason why the DRNs did not include functional sensorimotor networks could be that the stimulus is brief. Furthermore, DRNs entail structures in which activity dissipates later because the activity can reverberate through the SC (see Supporting Information Figure S1B–S1D). The unique components in the DRNs 2, 4, 6, 7, and 9 (see not highlighted components in Supporting Information Figure S1A) were correlated with none of the nine functional networks.

Similarly, the yellow motif one does not match any of the nine functional networks. The default mode and the salience networks have similarities at the isocortical level, match the teal motif two, and occur in most DRNs (11 out of 12 DRNs). The motif of the default mode network (and the salience network) appears to be the most sensitive because it was accessible from a large number of locations in the brain. It was the most flexible because it connects the different DRNs. The lateral network matches the orange motif four, which was less accessible because it was present in a small number of DRNs (i.e., 1, and 3 to 5). Similarly, the medial network matches the gray motif 3 (DRNs 8 to 12). In both cases, the motifs reflect the activity within one hemisphere (lateralized organization).

### Organization of Brain Activity Following the Sensory Pathways

To test whether the experimentally derived large-scale mouse brain network contains the structures and connections of sensory pathways, we extracted network descriptions (see Supporting Information Figure S7) of five sensory systems (auditory, the visual, the whisker, fore, and hind limbs). The networks are described according to the scientific consensus from the literature (see Materials and Methods). The analysis shows that long-range SC statistically well represents sensory networks. The brain areas in the sensory pathways (Supporting Information Figure S7) provide prior knowledge about the brain’s response consistency when stimulating precisely these areas. However, this is not trivial because the pathways are a small part of the large-scale brain network. The sensory pathways in Supporting Information Figure S7 represent that sensory processing follows specific paths of activations toward the primary somatosensory cortices. Activation of sensory processing can be ipsilateral to the sensory organ, such as for the whisker follicles up to the midbrain (see Supporting Information Figure S7D). The sensory processing usually crosses to the contralateral side of the sensory organ, indicating the integration of the left and right sensory organs of the same modality (see Supporting Information Figure S7). For instance, the auditory pathways show directed processing to the contralateral side (projections from the medulla to the pons). Then, the path continues to the midbrain, the thalamus, into the primary somatosensory areas (see Supporting Information Figure S7B).

### DRNs and Sensory Systems

We used the catalog of brain responses to examine those DRNs that respond to brain structures’ stimulation in the sensory pathways. We can thus trace the activity and dynamics in each of the sensory systems. Brain areas involved in sensory processing along the primary paths may be connected to other (secondary) brain areas supporting one or several functional networks.

### Primary Auditory Pathways

The catalog of DRNs and its stimulation sites (see Figure 2) responses were consistent and altered along the auditory pathways (bottom-up). Three DRNs hierarchically organized the formation from the cochlea to the primary auditory areas. From the cochlea up to the cortex through the medulla, the pons, the midbrain, and the thalamus:

1.  DRN number 5 (see Figure 2) due to stimulation in the medulla (DC, VC)
2.  DRN number 9 due to stimulation of the medulla (Tz), the pons (NLL), and the midbrain (BIC)
3.  DRN number 10 due to stimulation of the midbrain (IC) and the thalamus (MG)

### Primary Visual Pathways

The responses were consistent throughout the visual pathways and had the same DRNs (number 10 in Figure 2). The responses were consistent and altered along the pathways of the whiskers (bottom-up).

### From the Whisker Follicles to Somatosensory Cortices

Three DRNs hierarchically organized the formation from the whisker follicles to the barrel field in the primary somatosensory areas. From the follicles to the isocortex through the medulla, the pons, the midbrain, and the thalamus (abbreviations of brain areas are given in Supporting Information Tables S1 and S2):

1.  DRN number 6 (see Figure 2) due to stimulation of the medulla (SpVc, SpVi, SpVo) as well as the pons (PrV)
2.  DRN number 6 due to stimulation in the midbrain (SuCo) and the thalamus (LD)
3.  DRN number 8 due to stimulation of the two thalamic areas (VPM, Po)

### From the Limbs to the Primary Somatosensory Cortex

The response to stimulation of the two nuclei in the medulla involved in the sensory pathways of the limbs (CN, GN) was correlated and had the same DRN, number 6 in Figure 2 (abbreviations of brain areas are given in Supporting Information Tables S1 and S2). The responses to stimulation of the nuclei in the thalamus (VPL, VPM) differed and belonged to two DRNs with the number 8 and 7 (for the parvicellular part).

### Activated Structures and Their Activated Areas Along Sensory Pathways

The cerebellum was the most active brain structure, in particular after stimulation of the auditory, visual, and whisker pathways. In the case of the whisker pathways, the thalamus was similarly active as the cerebellum. The cerebellar activation was the strongest of all modalities and mainly focal after the auditory pathway stimulation (at the flocculus and the copula pyramidis). Significant activation of the isocortex occurred only upon stimulation of the auditory and visual pathways. The laterointermediate area (LI) in the isocortex, which is interestingly a visual area (Garrett et al., 2014), responded the most to stimulation of the thalamic visual nuclei (dorsal part of the lateral geniculate complex of the thalamus, left and right) and, albeit less, to stimulation of the auditory nuclei in the midbrain (the nucleus of the brachium of the inferior colliculus, BIC, left and right). Activity in the other brain structures was less significant, and the patterns were more scattered and complicated. Only the stimulation of nuclei in the limb pathways showed activity that was less distributed within the cerebellum, the thalamus, and the isocortex.

Areas in the hypothalamus were the most active ones throughout the stimulation sites and modalities. Their activation patterns were lateralized (in the first (dominant) component of a DRN). They showed activity contralateral to the stimulation sites in most of the cases (except vision, i.e., not lateralized). The most active areas were the intermediate part of the periventricular nucleus (PVi), the lateral mammillary nucleus (LM), and the arcuate nucleus (Arc). Table 4 ranks the activity of brain areas after stimulation of the pathways.

**Table 4.** Stimulation of the sensory pathways activated brain areas

Rank	Visual	Auditory	Whisker	Limbs
1	LI (isocortex)	Arc (hypothalamus)	PVi (hypothalamus)	PVi (hypothalamus)
2	Arc (hypothalamus)	DC (medulla)	S1, mouth (isocortex)	S1, lower limb (isocortex)
3	PVi (hypothalamus)	PVi (hypothalamus)	Arc (hypothalamus)	AN (medulla)
4		AN (medulla)	S2 (isocortex)	GN (medulla)
5		A1 (isocortex)	EC (isocortex)	NA (medulla)
6		LI (isocortex)		LI (isocortex)
7				Visc (isocortex)
8				LM (hypothalamus)

*Note*. Activated areas are ranked according to their strength of activity. Abbreviated brain areas are listed in Supporting Information Table S1.

### Symmetries in the Brain Responses

We found only weak stimulation asymmetries, which point toward homologous activation after left and after right stimulations. Activation was left/right stimulation-symmetric in the sense that the activity of the right areas after the stimulation of a left functional area (e.g., left LGN) corresponds to the activity of the left areas after the stimulation of the same right functional area (e.g., right LGN). Most of the activity after stimulation of the visual, auditory, whiskers, and limb pathways showed lateralized and left-right stimulation-symmetric patterns. A reason for this symmetry is that the tracer experiments compiled in the ABA mostly relate the spatial diffusion of the tracer to the injection site in the right brain. The SC was consequently left/right symmetric in the mouse model. The activity mostly cumulated either ipsilaterally or contralaterally to the stimulation site. However, only in the medulla, we found nonlateralized symmetries across all modalities. Nonlateralized left-right stimulation-symmetric activation patterns also occurred in the cerebral nucleus for stimulation of the auditory pathways and the pons for stimulation of the whiskers pathways. The limb pathways showed most nonlateralized symmetries across structures (olfactory areas, hippocampus, cerebral nuclei, pons, and medulla).

The majority of activation patterns in the subisocortex were significantly bilateral symmetric (activity on the left side was similar to the ones on the right). In contrast, patterns on the isocortex were more variable for stimulation of all brain areas involved in the sensory pathways (across all modalities and left and right areas). Whether the activation patterns on the isocortex showed no symmetry (sign for lateralization), antisymmetry (left/right inverted activity), or symmetries (bilaterally symmetric) was specific to the stimulation location in the auditory, visual, whisker and limb pathways.

### Comparison of In Silico and In Vivo Brain Dynamics After Stimulation

**Figure 3.**
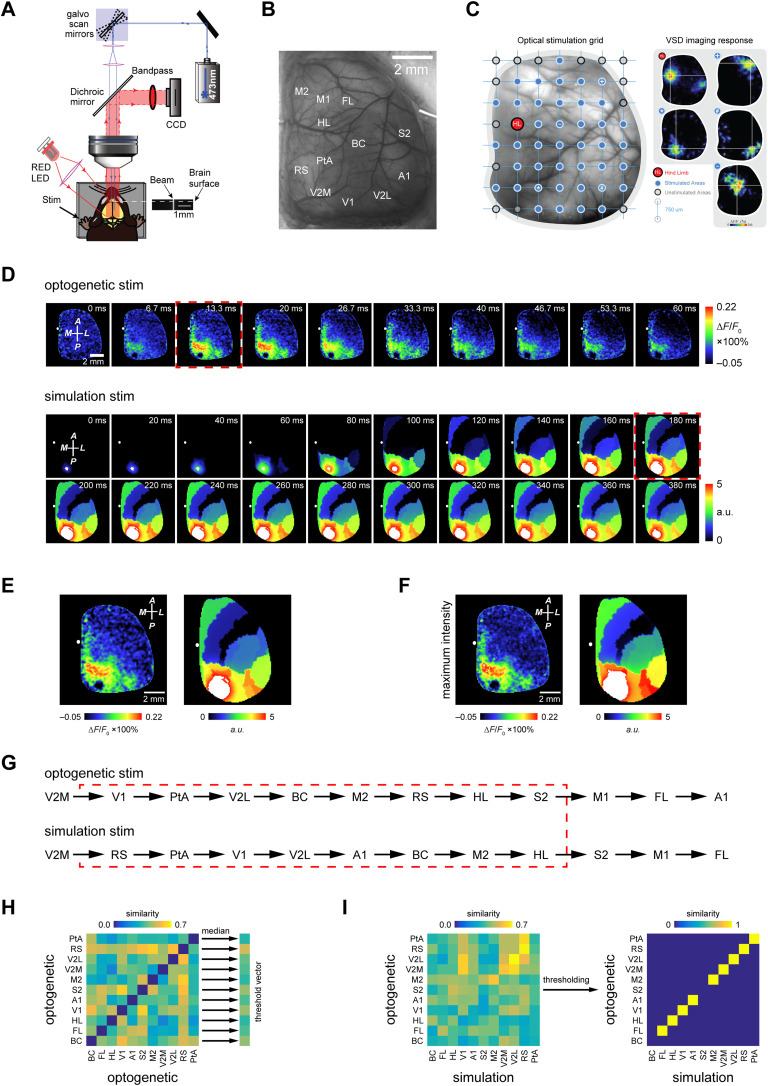
Comparing the empirical and simulated stimulus-induced spatiotemporal pattern of activity in the neocortex. (A) Schematic of the experimental setup. This setup consists of a 473-nm laser generator whose beam is directed by the galvo scan mirrors to a specific point on the cortical surface. By changing the configuration of the galvo scan mirrors, we can change the coordinates of the stimulated point. The CCD camera captures the voltage activity of the brain. (B) An example of a cranial window over the mouse cortex prepared for VSD imaging. Neocortical regions are labeled according to the Paxinos mouse brain atlas based on their coordinates to the bregma, marked by a white circle. ABA equivalent of these regions is listed in Table 5. (C) An example of the grid points mapped on the neocortical surface by the galvo scans mirrors. The laser can stimulate each one of these points. Examples of the voltage activity right after stimulation of some of these points are presented on the right. Comparing the empirical and simulated stimulus-induced spatiotemporal pattern of activity in the neocortex. (D) Spatiotemporal pattern of voltage activity after optogenetic (top) and simulated (bottom) stimulation of V2M. (E) The enlarged frames, specified by a red rectangle in panel D, juxtaposed for qualitative comparison. (F) Spatial distribution of the poststimulus maximum voltage activity. (G) Temporal order of activation of neocortical regions after optogenetic (top) and simulated (bottom) stimulation. Activation time stamp is when the poststimulus voltage level of a given area surpasses 20% of its peak activity. (H) Similarity matrix consisting of the similarity values between all the pairs of the temporally ordered sequences of activated regions with length 8 (see the red dashed rectangle in panel G) induced by optogenetic stimulation of neocortical regions. The similarity value between two sequences was calculated in three steps: (1) The first element of each sequence is omitted, and then the nonoverlapping elements of two sequences were made the same. For example, GABCD and HCFEA became ABCD, and CBDA (the identity of the elements chosen to substitute the nonoverlapping elements must be different from the overlapping elements). It ensures that the two sequences are made of the same elements. (2) The total number of possible transpositions normalized the number of transpositions needed to convert one sequence to another. That gives a measure of the distance between the two sequences. (3) Thus, one minus distance gives a measure of similarity between the two. For example, the similarity value between ABCD and CBDA is 1 − 2/3 = 1/3). The similarity value is multiplied by the ratio of the number of overlapping elements between the two original sequences and the total length of sequences. For example, the similarity value between sequences ABCD and CFEA is 1/2 1/3 = 1/6. The values on the diagonal of the matrix (self-similarity, which is always 1) were purposefully removed. The median of each row gives an expected value for the similarity of the spatiotemporal patterns of activity induced by stimulation of the corresponding and rest of regions. (I) (left) The same as panel H but for similarity values between empirical versus simulated spatiotemporal patterns of activity induced by focal stimulation of different neocortical regions (see the red dashed rectangle in panel G); (right) the similarity matrix between empirical and simulated patterns was thresholded and binarized using the threshold vector generated in panel H. To highlight how similar the empirical and simulated patterns were, only the values on the diagonal are presented.

**Figure 4.**
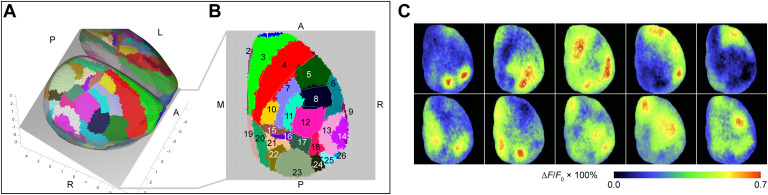
Modeling the focal plane for voltage-sensitive dye (VSD) imaging. (A) 3D geometric model of the mouse brain reproduced the camera setup in the mice experiments. The focus was first on the right isocortex surface and then adjusted 1 mm inside the cortex so that the depth of field covers most of the right isocortex (for the experimental setup, see Figure 3A). The consequence is that the focal plane cuts through the right isocortex (see the gray plane in panel A) and records brain activity from a wide field of the isocortex. (B) The visible neural masses were projected onto the focal plane, using the normal vector of the focal plane (i.e., assuming an infinite focal length). The modeled focal plane is sampled by an array of 128 × 128 pixels by the camera sensor used in the experiments for the VSD imaging recordings. Because each brain area in the isocortex comprises several neural masses, areas are colored in both panels A and B. Areas captured by the VSD imaging are numbered in panel B and listed accordingly in Table 5. The labels A, P, L, R, M denote locations: (A)nterior, (P)osterior, (L)eft, (R)ight, and (M)edial. (C) Experimental VSD imaging of spontaneous brain activity recorded in isoflurane-anesthetized mouse shows spatial organization (here 10 typical patterns) demonstrating the qualitative correspondence of experimental and modeled VSD imaging purely based on the ABA parcellation. Note that the (secondary and primary) motor areas (areas 3 in green and 4 in red in the panels A and B) and somatosensory areas such as the barrel field (area 12 in magenta in the panels A and B) represent spatially extended and functionally undivided areas (e.g., by topographic maps) in the ABA. VSD imaging can indicate activity patterns that are finer grained than the used ABA division of the isocortex into brain areas in the model. Snapshots of the experimental VSD imaging of spontaneous activity in panel **C** were taken and adapted with permission from Mohajerani et al. (2013) (Supporting Information Video S2).

We have, so far, shown that our model is capable of generating brain-wide spatial patterns resembling previously identified functional networks. To test our model in generating realistic brain activity, we compared simulated and empirical dynamics after the focal stimulation of isocortical regions. We used in vivo wide-field VSD imaging combined with optogenetic focal stimulation (Figure 3A). This technique allowed us to capture brain activity over a large portion of the isocortical mantle (Figure 3B). In contrast, an arbitrary point on the isocortical surface was stimulated (Figure 3C). The *observer system for VSD imaging* linked the mouse brain model (mean postsynaptic potentials) to the 2D VSD images (see Figure 4, Table 5, and Materials and Methods for further details). For comparing model and in vivo data, we used the exemplary model configuration of 500-*μ*m short-range SC with a ratio of 40% short-range SC to 60% long-range SC (i.e., *α* = 0.4) and the stimulation period of *η*^−1^ = 13 ms.

**Table 5.** Brain areas captured in vivo by the voltage-sensitive dye imaging and in the virtual mouse brain model

No.	Areas captured by the VSD imaging	No.	Areas captured by the VSD imaging
1	Frontal pole	14	Primary auditory area, *A1*
2	Anterior cingulate area, dorsal part	15	Primary somatosensory area, trunk
3	Secondary motor area, *M2*	16	Anterior area, *PtA*
4	Primary motor area, *M1*	17	Rostrolateral visual area
5	Primary somatosensory area, mouth	18	Anterolateral visual area
6	Supplemental somatosensory area, *S2*	19	Retrosplenial area, *RS*C, dorsal part
7	Primary somatosensory area, upper limb, *UL*	20	Retrosplenial area, *RS*C, lateral agranular
8	Primary somatosensory area, nose	21	Anteromedial visual area
9	Ventral auditory area	22	Posteromedial visual area, *V2M*
10	Primary somatosensory area, lower limb, *HL*	23	Primary visual area, *V1*
11	Primary somatosensory area, unassigned	24	Lateral visual area, *V2L*
12	Primary somatosensory area, barrel field, *BC*	25	Laterointermediate area, *LI*
13	Dorsal auditory area	26	Temporal association areas

*Note*. The numbering (No.) of the 26 listed areas (out of 84 isocortical areas) in the isocortex (total number of 512 brain areas), captured by the voltage-sensitive dye imaging technique, corresponds to the labels in Figure 4B.

In contrast to the model parameter exploration, we adjusted the size of the stimulation focus to mimic the laser stimulation with a beam diameter of 360 *μ*m on the surface in the center of isocortical areas. We observed a qualitative similarity between the spatial extent of simulated and empirical activity patterns. The patterns were evoked by the focal stimulation of the different isocortical regions, including retrosplenial (RS) and visual areas (see Figure 3D and E, and Supporting Information Figure S8 and Figure S9). This similarity became more evident by comparing the pattern of the spatial distribution of maximum activation across neocortical regions after focal stimulation (see Figure 3F and Supporting Information Figure S8 and Figure S9). The spatial organization of these patterns was not trivially linked to the location of the stimulation focus and showed reproducible large-scale activation across the imaged cranial window. Both empirical and simulated data suggested that SC could predict the brain activity evoked by the focal stimulation of different regions. For example, focal stimulation of the RS cortex led to the activation of the visual and midline areas, which showed secure connections to the RS cortex.

Moreover, the temporal order of the activation of isocortical regions evoked by in vivo and in silico focal stimulation showed a detectable level of similarity (Figure 3H and I; Supporting Information Figure S1; Table 6 and Table 7). To measure the similarity between two ordered sequences of activated regions (Figure 3G and Supporting Information Figure S8, Figure S9, Figure S11, and Figure S13), the distance between the two sequences was first calculated using Kendall’s tau distance (Kendall, 1938). Then, one minus the distance between the two defined the unnormalized similarity between the two sequences. Similarity values were normalized. For that purpose, we multiplied each similarity value with the proportion of the overlapping elements out of all elements in each sequence (see Materials and Methods for a detailed description of calculating similarity values). To determine what range of normalizing similarity values (similarity value for short) between in vivo– and in silico–ordered sequences is considered meaningful, we calculated the similarity values between all possible pairs of ordered sequences generated by in vivo optogenetic stimulation of isocortical regions (Figure 3H). The median of all similarity values, associated with all the pairs consisting of a given isocortical region and all other regions, was considered as a threshold for evaluating the meaningfulness of similarity values between in vivo and in silico patterns of activity evoked by stimulating that region (Figure 3H). This analysis revealed that the model could generate stimulus-induced activity patterns similar to those evoked by optogenetic stimulation, that is, the case for the majority of the imaged regions—in particular the retrosplenial (RC), the posterior parietal (PtA), and the associational visual areas. These results indicate that our model assumptions can relatively explain the flow of information in some isocortical subnetworks. We want to emphasize that we did not fit the network model on the empirical data. We repeated the same analysis for two larger threshold values (75th and 90th percentiles of in vivo/in vivo similarity values). The results still show a considerable number of regions, for which the in silico/in vivo similarity passes the threshold (Supporting Information Figure S14 and S15).

**Table 6.** Temporally ordered activated neocortical regions in response to focal optogenetic stimulation of different region

**BC**	**FL**	**HL**	**V1**	**A1**	**S2**	**M2**	**V2M**	**V2L**	**RS**	**PtA**
M2	M1	S2	M2	PtA	PtA	PtA	V1	PtA	PtA	V2M
RS	S2	M2	RS	V2L	M1	M1	PtA	V2M	V2M	V1
PtA	M2	PtA	PtA	M1	M2	BC	V2L	V1	M2	V2L
V1	V2M	M1	V2M	HL	V2M	FL	BC	M2	V1	HL
M1	BC	FL	V2L	S2	V2L	V2L	M2	RS	V2L	S2
HL	HL	A1	BC	M2	BC	HL	RS	BC	M1	M2
V2M	PtA	RS	S2	V2M	HL	S2	HL	S2	HL	RS
V2L		V2M	M1	RS	FL	V2M	S2	HL	S2	BC
FL		V2L	HL		A1	V1	M1	A1	A1	A1
S2					V1	A1	FL	M1		FL
A1					RS	RS	A1			M1

*Note*. The first row of the table represents the stimulated regions, and the other rows in each column represent the sequence of activated regions sorted by their activation latencies from earliest to latest.

**Table 7.** The temporally ordered activated neocortical regions in response to simulated focal stimulation of different regions

**BC**	**FL**	**HL**	**V1**	**A1**	**S2**	**M2**	**V2M**	**V2L**	**RS**	**PtA**
V2L	HL	FL	V2L	V2L	BC	M1	RS	V1	V2M	V2L
PtA	M1	PtA	V2M	BC	FL	HL	PtA	A1	PtA	BC
FL	BC	M1	PtA	V2M	M1	FL	V1	PtA	V2L	A1
S2	S2	A1	RS	PtA	HL	PtA	V2L	V2M	V1	V2M
HL	PtA	M2	A1	S2	A1	S2	A1	RS	HL	RS
A1	M2	BC	BC	V1	PtA	BC	BC	BC	M2	HL
M1	A1	V2L	HL	HL	M2	V2L	M2	S2	M1	V1
V1	V2L	RS	S2	FL	V2L	A1	HL	HL	BC	FL
M2	V2M	S2	M2	M1	V2M	RS	S2	M2	S2	M1
V2M	RS	V2M	M1	RS	V1	V1	M1	M1	A1	M2
RS	V1	V1	FL	M2	RS	V2M	FL	FL	FL	S2

*Note*. The first row of the table represents the stimulated regions, and the other rows in each column represent the sequence of activated regions sorted by their activation latencies from earliest to latest.

We conclude that we observed a reasonable similarity between the simulated and empirical patterns evoked by stimulating certain isocortical regions.

## DISCUSSION

Brain network dynamics can be *systematically* explored only in silico. To explore mouse brain dynamics in silico, we built a high-resolution whole mouse brain network model. Our model structure originates from ABA, comprising viral vector tracer studies. We investigated the network behavior by assuming simple oscillatory but canonical dynamics at each location in the brain model. Only the SC from the ABA differentiated the local dynamics. We simulated the whole-brain mouse model on a computer by using the neuroinformatics platform TVB. The emergence of specific functional networks in the simulated brain responses is the main result of the in silico exploration. To test whether the mouse brain activity could be exclusively inferred from the underlying connection topology, we performed a graph-theoretic analysis of the model structure. The results showed that the overall performance of the 13 graph-theoretic measures was rather weak. The best prediction of the simulated brain responses to focal stimulation and the extracted DRNs resulted from comparing the activity’s rank with the rank of SC strengths of the direct projection from the stimulation site. The high correlation observed indicates that energy induced by the stimulation spreads into areas of the brain that were directly connected to the stimulation site. The rank correlation was weak but significant for most connectivity parameter configurations. Then, we compared measured and simulated brain activity by using the observer model for (virtual) VSD imaging.

In our model for exploring the mouse brain dynamics in silico, we systematically varied three model parameters, namely:

1.  the ratio, to which extend brain areas can interact over short and long ranges,
2.  the range of short connections in the isocortex, and
3.  the location of focal stimulation.

The extent to which information is processed over short- or long-range SC is unclear. The results show that both SC types of short- and long-range connections in the mouse model act as spatiotemporal filters of local dynamics in the emergent functional network. Our results (Supporting Information Figure S6) do not support evidence for a reorganization of dynamics due to interplay between both types of SC in the mouse as opposed to human models (Spiegler et al., 2016). We analyzed the brain responses to stimulation. The focal stimulation is closely related to direct electrical, sensory, and optical stimulation. We identified consistent spatial patterns of mouse brain activity in response to stimulation called the DRNs by using dimension reduction methods. We observed some of the previously and experimentally known functional networks, including the salience and default mode networks, in the DRNs.

Interestingly, the salience and the default mode networks spanned several DRNs, indicating the salience and default mode networks are junctions for functional networks. Furthermore, we investigated the spatiotemporal organization of brain activity in sensory pathways. We considered the following sensory pathways: the auditory, the visual, the whisker, and the limb somatosensory pathways. With the developed large-scale mouse brain network model, we had, for the first time, the chance to systematically investigate sensory pathways and, primarily, the processing of sensory data. In the end, we showed that the in silico brain responses, to some extent, resemble in vivo brain dynamics after stimulation. In the following, we discuss the results in more detail.

### Modeling Results

The structure of the model (Figure 1), based on the ABA comprising viral vector tracer studies, was co-registered with the anatomy of the mouse brain (see Oh et al., 2014). We spatially sampled the brain volume given by the ABA and obtained a network model composed of 512 distinct brain areas. The model incorporates the smooth surface of the isocortex that we reconstructed from the ABA by using a regular mesh of 27,554 triangles with 41,524 edges between 13,972 vertices (Supporting Information Figure S2) and the spatial extent of the 84 cortical areas given by the ABA. The 512 distinct brain areas contain the 84 isocortical areas (42 per hemisphere) and 428 nonisocortex areas (see Table 1 for the division and Supporting Information Table S1 and Table S2 for a complete list of area names). We lumped each of these 428 subisocortical areas in the model to a point in physical space (i.e., centroid). From these numbers, it is evident that the isocortex constitutes only a small proportion of brain areas considered in the model (and in the ABA).

Moreover, the wide-field VSD imaging, used in this study, captures activity from only 26 isocortical areas in the ABA. Thus, we observed the activity of a small fraction of interconnected areas (26 observables vs. 512 brain areas in the model). Here the spatial resolution in the model must coincide with the resolution of the measurement. Although the resolution of brain geometry (e.g., the isocortical mesh) agrees with that of the measurements (e.g., VSD imaging), quality of the modeling highly depends on the division of the brain into areas (parcellation) and the resolution and reconstruction of connections (Proix et al., 2016). In the version of the ABA that we have used to construct the model, for instance, the barrel field of the primary somatosensory areas is summarized in one area (see Supporting Information Table S1). The model can be improved by using not only the new ABA model (Knox et al., 2019) but also by incorporating other databases (e.g., Dorr et al., 2008; http://www.mouseimaging.ca/research/mouse_atlas.html; www.mouseconnectome.org).

The experimental data collected by the ABA was the base for the model structure (i.e., geometry and connectivity) and the division of the high-resolution model into brain areas. The local dynamics, though, were less grounded since less data is available from large-scale brain measurements. Electrophysiology allows recording local activity (in a distinct brain structure) from a limited number of sites. Such data do provide information about characteristic changes in space and time. However, they do not clarify if an activation pattern is related to function or dysfunction (e.g., epileptic discharges) originated locally or emerged throughout the network’s interactions. Brain stimulation techniques are here an appropriate method to infer local characteristics (e.g., cortical excitability) by probing the formation of characteristic patterns in the brain activity (e.g., spreading and dissipation of activity from the stimulation site).

### Modeling Brain Dynamics and Limitations

The large-scale network model contains the anatomy of the mouse brain, the geometry of the cortices, the brain areas, and their axonal connections. We performed simulations assuming that the structure of the mouse brain defines its function and dysfunction (and vice versa). Both of them find reflections in brain signals such as electrophysiological recordings and VSD imaging.

For each location in the network model, we used simple dynamics. Simple dynamics (e.g., planar systems can show smooth and rhythmic responses to stimulation) are often building blocks of systems with higher complexity (e.g., Jansen–Rit model within specific ranges for each parameter; see Spiegler et al., 2010, 2011). However, rich emergent spatiotemporal behavior (e.g., the occurrence and propagation of epileptic discharges across distant brain areas (Jirsa et al., 2014; Proix et al., 2014), or network dynamics with nontrivial connectivity and time delays (Jirsa et al., 1995; Feng et al., 2006; Jirsa, 2007; Pinotsis et al., 2013; Spiegler et al., 2016) further illustrates the complexity of systems. Therefore, it cannot be embedded in simple systems. The strategy to build models as simple as possible and as complex as necessary provides a discussion about the integration and emergence of function and dysfunctions in the brain, especially at the large-scale of entire brains (for a review, see Deco et al., 2011; Breakspear, 2017).

The rhythmic activity allows for effective transmission of information via the connections between brain areas. For this purpose, the rhythmic activity needs to be fast (e.g., faster than 40 Hz) or the conduction speed needs to be slow (e.g., less than 1 m/s) in consideration of the length of axonal fibers connecting brain areas (e.g., mean length of about 6.25 mm). We assumed a natural frequency of 42 Hz at each network location and conduction speed of 1 m/s, both within biologically plausible ranges. The axonal conduction speed is determined by the axonal diameters as well as the degree of myelination, both of which vary throughout the mouse brain (Wang et al., 2008; Foran & Peterson, 1992). Compared to larger mammalian brains, including human brains, axons are less myelinated and are shorter in the mouse brain (see, e.g., Wang et al., 2008). The axonal conduction speed in mice is in the range of 0.2 m/s (Wang et al., 2008) for unmyelinated axons with an average axonal diameter of 0.3 μm (Braitenberg & Schüz, 1998). The axonal conduction speed for myelinated axons is in the range of 0.3 m/s and 3.5 m/s (Wang et al., 2008; Waxman, 2012; Salami et al., 2003). Axonal length and conduction speed both cause time delays in the transmission of information processing. Myelinated axons are slower in mice and more similar to unmyelinated axons in human brains (Wang et al., 2008). The ratio of myelinated to unmyelinated axons is smaller in mice than in human brains (Wang et al., 2008). Also, myelinated axons are shorter in mice brains than in human brains (Wang et al., 2008). Note that the injection techniques underlying the ABA do not provide an estimate of the axonal lengths. The Euclidean distances between brain areas could be used as a proxy to estimate axonal lengths. Diffusion tensor MRI data are useful datasets to estimate the axonal tracts and to compile a connectivity matrix.

In contrast to the ABA, the diffusion tensor MRI does not provide directionality but mouse individual connectivity information. With the availability of diffusion tensor MRI data of mice, the ABA connectivity could be co-registered with the diffusion tensor connectivity, and thus provides information about axonal lengths. To the best of our knowledge, however, neither a complete nor an extensive classification is currently available for the characterization of all 512 considered brain areas in the model.

By a simple local dynamic model, we probed the experimentally derived large-scale brain network to produce functionally relevant organizations in the activity induced by stimulation. If the model equipped with this simple dynamic does not show functionally relevant organizations in the simulated brain activity, then the model lacks relevant complexity. When the experimentally derived large-scale brain network activation misses essential features (perhaps the average brain structure is not sufficient and averages out essential components), better data (e.g., better estimation for the connectivity strengths) will be required. The behavior in the large-scale brain signals (e.g., M/EEG and functional MRI) associated with brain function and dysfunction is perhaps epiphenomenal at the large-scale of the brain. The behavior could originate from processes on the micro- and mesoscopic scale (rather than through the interaction of brain areas) projecting onto the large-scale of the whole brain and its functional measurements.

Another point is the local dynamics. The local model for the simple dynamics that we used here includes a surrogate parameter for the ratio of excitation and inhibition. However, this model is generic and does not represent a particular local structure. If this model is not sufficient, biologically informed models are available. The local dynamic models can be biologically informed about the local circuitry and specific activities (e.g., certain rhythms). Local circuitries are sketches of characteristic elements (e.g., projection neurons, interneurons) and their connections involve in relevant processes (e.g., excitation/inhibition) based on morphological and physiological arguments. Using such biologically informed models increase the repertoire of dynamics at network locations by their local circuitry, which are thus meso- and microscopic descriptions. To the best of our knowledge, however, neither a complete nor an extensive classification is currently available for the characterization of all 512 considered brain areas in the model (e.g., to excite or to inhibit synaptically connected area).

### DRNs and Functional Networks

We performed a systematic exploration of brain dynamics via focal stimulation to extract the DRNs. The local model dynamics and the structural connectional topology determined the DRNs. The interplay of these factors imposes conditions on the dynamic repertoire of a given network, independent of the details of how these factors are physiologically realized. To be specific, there is a multitude of ways to generate a particular behavior of a neural population (Marder & Taylor, 2011), such as an oscillation. Once such behavior is established, it communicates via the connectivity with other regions. The actual physiological underpinnings then play no role unless new factors such as spike rate adaptation arise. From this perspective, the DRNs fundamentally reflect the functional dynamic repertoire of a network given particular connectivity.

Using the mouse model, we extracted the characteristic brain responses, that is, DRNs and the motifs of activity pattern linking the DRNs. We found five motifs of characteristic activity patterns connecting the DRNs. The recurrence of a motif in several DRNs suggests that the brain can phrase more than one motif (thus more than one DRN) at a time, endogenously and exogenously by stimulation. Comparison of the motifs and experimentally known functional networks suggest the role of the networks in dealing with stimuli. Four of the five motifs in the DRNs matched four of the nine functional networks. We found the default mode network (together with the salience network), the most dominant motif that connected almost all DRNs. The DRNs do not include sensorimotor networks. One reason for this could be that the DRNs entail structures in which activity can reverberate through the SC and dissipate later (see Supporting Information Figure S1B–D). In other words, the early stages of the brain response, which are not captured by the DRNs, reflect the sensorimotor networks. The catalog of responses and the mapping to stimulation gives the prospect of a coordinated interaction with functional networks by using different types of stimulation (e.g., sensory and transcranial).

The used local model is simple and appropriate for investigating the large-scale spatiotemporal organization of brain responses, which includes the scope of this work. As a consequence of the monorhythmic behavior and the uniform parameterization of the local dynamic model, the simulated network dynamics show limitations in describing the various rhythms involved in the brain organization (i.e., neuronal firing and brain rhythms alpha to gamma). To describe the broadband frequency behavior (as observable in EEG), the network model has to allow the generation of more complex activity, potentially subject to subsequent studies. The modeling is challenging, and it goes hand in hand with the debate about the generation of brain activity such as rhythms and especially its spatial extent. The risk in this kind of modeling work based on large-scale data (as with modeling based on microscopy data, see Marder & Taylor, 2011) lies in the overfitting (additional state variables increase the degree of freedom and may increase model complexity). One approach would be to use the same simple local dynamic model for each location in the mouse brain network as we have done in this work but then parameterize local dynamics differently to account for the spatial differences in natural frequencies.

The applied measures to the SC evaluate the mapping of monosynaptic connections between neurons in a structure and the target neurons in the brain. Therefore, it is not surprising that the measures were insufficiently able to predict the simulated brain activity following stimulation. It becomes evident, considering the emergence of DRNs. The dissipation of brain activity from several stimulation sites into one of the 12 DRNs required more than one connection, simply because of distances. The DRNs also covered the entire brain of the mouse (see Figure 2). Polysynaptic pathways connected stimulation sites and the activated DRNs, comprising patterns of coactivated brain regions.

Recursively applying the SC renders polysynaptic connectivity pathways as follows. The SC (monosynaptic connections) maps the activity from the stimulation site to targeted brain areas. In subsequent steps, the SC maps activated areas to targeted areas of the brain. The iteration forms pathways with polysynaptic connections.

That is what happens in dynamic models. A crucial point of the in silico modeling in this work is the nonlinear (local) dynamics involved in the mapping of the SC onto itself at all times. While the activity patterns of the DRNs reflect functional connection topologies, the polysynaptic connectivity pathways between a DRN and its stimulation sites create a causal relationship, known as effective connectivity, and for which axonal transmission times are crucial. Measures of effective and functional connectivity (EC and FC) in empirical data assume linear dynamics (see, e.g., Friston, 2011). In contrast, the nonlinear dynamics allow the model to perform oscillations and more complex behaviors requiring higher order techniques for model inversion (e.g., Monte Carlo Markov Chain methods; Hashemi et al., 2020). Measures of FC dynamics have characterized the alteration of the functional connectional topology at rest and the emergence of RSNs (e.g., Hansen et al., 2015; Allen et al., 2012). Here, stimulation paradigms provide a causal link between brain activity and stimulation. For a current review of the literature on the relationship between structure and function in the mouse brain in conjunction with optogenetic stimulation, we would like to refer the interested reader to the paper by Snyder and Bauer (2019).

Comparisons of FC derived from resting-state fMRI of mice and SC have been only partially successful, for example, for cortico-cortical and cortico-striatal regions, which have been related to monosynaptic SCs (see, for instance, Grandjean et al., 2017). The synchronous activity in homotopic cortical areas mainly causes the bilateral symmetry in the FC and may reflect monosynaptic, transcallosal SCs, or coordination by subcortical structures (Snyder & Bauer, 2019). Our modeling results indicate that DRNs with bilateral symmetric activity patterns involve stimulation of mostly deep (subcortical) brain structures (e.g., medulla, cerebellum, midbrain, and pons in DRN 5; see Figure 2). In contrast, DRNs with bilaterally asymmetric patterns required stimulation of the cerebrum and thalamus. The DRN with number 10, for instance, needed stimulation in the isocortex and the thalamus (see Figure 2).

EC after optogenetic stimulation has been reported to match SC more closely than resting-state FC, using fMRI (Snyder & Bauer, 2019). According to our model, the analyzed hemodynamic responses should reflect the early activity. Also, the captured brain activity should have passed monosynaptic connections from the stimulation site. The model would otherwise predict that the EC seizes polysynaptic connectivity pathways (later response components), which does not necessarily match the SC, as in the case of the DRNs.

To estimate EC and perform hypothesis testing, Ryali et al. (2016) used an autoregressive model. This modeling is based on the empirical time series and can provide an interpretation of network structures since they can distinguish between intrinsic and context-specific modulatory causal interactions in latent signals. Autoregressive models, as used by Ryali et al., do not comprise biologically informed local dynamics but assume local linear dynamics. Besides, the intrinsic connections are subject to the model inversions. In contrast, our in silico modeling is based on the SC collected by the ABA (specifying causal interactions and transmission delays) and works in conjunction with a nonlinear local dynamic model that enables oscillatory behavior. Also, this in silico modeling allows for systematic exploration as well as hypothesis testing. An advantage of modeling the mouse brain is that its global dynamics are more readily perturbed and observed, permitting better comparison between in silico and in vivo. Hence, connectome-based dynamical modeling has the potential to contribute significantly to the field, particularly given increasing usage of wide-field imaging of the mouse brain.

### Sensory Pathways

We investigated the DRNs that were responsive to the stimulation of areas involved in sensory processing based on the catalog of brain responses. Such investigations are possible in silico but hard to accomplish for an individual brain in vivo. Based on textbook descriptions (e.g., Watson et al., 2011), we compiled networks of sensory pathways (see Supporting Information Figure S7). We found these networks back in the SC collected by the ABA and are thus part of the mouse brain network model. However, the ABA needs refinements to distinguish lower and upper limb (especially hind and forelimb); see Supporting Information Figure S7E. Nor does the ABA include a division of the barrel field of the primary somatosensory areas. The networks in Supporting Information Figure S7 indicate that information could flow to areas that are not necessarily the primary sensory areas of the isocortex. For instance, the hypothalamus and the midbrain are targets of the retinal ganglion cells (see Supporting Information Figure S7A). The midbrain nuclei are related to whisker movements (see Supporting Information Figure S7D). These connections are well studied and usually not discussed in textbooks sections about sensory processing. As a result, the visual and the whisker systems meet in the sensory-related superior colliculus regarding eye and whisker movements. It is worth mentioning that the model is agnostic about the (sensory) information, meaning that nuclei may be activated, but the object of processing (information) is less defined. The vast amount of studies about the physiology of nuclei allows attaching meaning to the modeling results. For instance, the hypothalamic nuclei and nuclei in the midbrain receive input from the retina. The nucleus of the optic tract, the olivary, and the posterior pretectal nucleus are the relevant nuclei in the midbrain. However, they are not directly part of the visual pathways, running from the retina to the primary visual cortices (see Supporting Information Figure S7A). The hypothalamic nuclei play a role in the circadian timing system, and the nuclei in the midbrain are known to be involved in eye movement coordination and reflexes.

Specific patterns in the auditory and visual pathways belong to the same cluster. The spatial proximity of relevant nuclei may cause this result. In the midbrain, the inferior colliculus (IC) and the sensory-related superior colliculus are neighboring. The resolution of the mouse tracer studies may be low because of leakage.

There is also overlap between whisker and limb pathways. An explanation is that the barrel cortex was not well separated. The primary somatosensory area distinguishes seven areas (see Supporting Information Table S1), including the barrel field (area 5), the lower limb (area 6), and the upper limb (area 8). Consequently, the mouse brain’s parcellation might be misleading because it stemmed from an average mouse brain compiled by many different brains (genetically the same mouse type).

Ventral posteromedial nucleus, ventral posterolateral nucleus, and posterior complex are neighboring areas. All are part of the posterior nuclei of the thalamus. The grouping may indicate uncertainty and limitation of the data. The spatial proximity and the local diffusion of the tracer during the measurements are challenges for the agglomeration of various experiments.

### Comparison Between the Mouse Brain Dynamics Model with Human Brain Dynamics Model

In this study, we adapted our published in silico human focal stimulation protocol to develop a mouse model to explore mouse brain dynamics. The two models are quantitatively comparable because the number of network nodes (locations) used to describe the brain anatomy is similar (16,500 in human vs. 14,000 in mouse). However, there are differences in the details. Brain size differs as well as the number of brain areas in the subcortex. The human brain model considered 76 cortical areas and 116 subcortical areas, which are all thalamic nuclei. Our mouse brain model considers 84 isocortical areas and 428 subisocortical areas, including 38 thalamic nuclei. The mouse brain model used in this study is most detailed in the nonisocortical structures, namely cerebellum, medulla, pons, midbrain, hypothalamus, thalamus, and cerebral nuclei. Nevertheless, it does provide an excellent validation framework for the large-scale brain network modeling approach.

One of the main questions in the brain network study is, to which extent information is processed via short- and long-range connections. Results in human studies show that short-range connectivity is relevant for explaining experimentally known functional networks. By varying the spatial extent of short-range connections and the ratio of short- to long-range connections, we observed a reorganization of the ss-DRNs and the brain dynamics in humans. In the mouse model, we did not find a reorganization of brain dynamics due to changes in the connectivity. Although the emergence of the ss-DRNs is sensitive to the transmission time delays between brain areas, the interplay of the two connectivity types appeared to be less critical in the mouse model than in the human model. The findings indicate that the used short-range SC in the isocortex is not sufficient for observing reorganizations of functional networks due to changes in the SC. The model suggests that 12 DRNs orchestrated the mouse brain dynamics around five motifs (see Figure 2). These DRNs were reasonably stable for a variety of mixture of short-range and long-range connections (see Supporting Information Figure S6).

Basic functional connectional topology is conserved across mice, rats, primates, and humans (van den Heuvel et al., 2016; Betzel & Bassett, 2018, for a review, see Snyder & Bauer, 2019). In this work, the motifs of the DRNs in both the mouse and the human model reflect functional networks. Whereas the DRNs in human span a variety of functional networks (e.g., default mode, memory, attention), the DRNs in mice mainly consist of functional networks at rest such as salience, default mode, medial, and lateral networks and less the sensorimotor networks (only the network of the frontal eye field).

The application of brain stimulation techniques is limited in humans to transcranial and sensory stimulation and intracranial stimulation, specifically in patients. In animals, a battery of interventions is available such as optogenetic tools and electrophysiology. These tools make the validation of models feasible. Besides, these mathematical models enable knowledge transfer from animals to humans. That is why modeling and in silico simulations are so important.

### In Vivo Sensory and Focal Stimulation in the Mouse Brain

Stimulation paradigms are critical for the validation of any model because conceptually, they take the system out of its attractor states and allow the sampling of the dynamic neighborhood of attractors. Thus, such paradigms significantly yield more predictive information than any resting-state paradigms do.

Although we did not fit our model to the empirical data, our model could somewhat replicate the stimulation-induced brain activity captured by VSD imaging. The simulated patterns evoked by the focal stimulation of primary barrel and secondary somatosensory cortices, compared with other considered areas, showed a low level of similarity to their in vivo counterparts. It could be due to some inaccuracies reported in the ABA. For example, a recent study (Knox et al., 2019) has shown that there could exist a significant error in the strength of axonal projections to primary somatosensory and, more importantly, from the thalamus to other regions. One explanation could be an inaccurate representation of the projection strength of the thalamus and the neocortex. The same reason could cause a lower level of similarity observed between empirical and simulated data. We believe that by incorporating a more accurate SC of neocortical networks, we can achieve a more realistic model that can produce patterns of activity resembling those seen in the empirical data.

Beyond connectivity, there are other significant simplifications present in the current network model affecting its validity, when confronted with empirical data. The most obvious one, from the network perspective, is the assumed homogeneity of local network dynamics. It is well established that local brain region dynamics vary across the network. Variations can occur differently. For instance, in terms of excitability, physiological rhythms measured in intracranial human EEG vary across brain regions (see Bartolomei et al., 2017). The intrinsic brain frequency varies and forms the posterior to the anterior gradient of eigenfrequency ranging from 10 to 40–50 Hz (e.g., Rosanova et al., 2009). The rhythmic organization can change and show such brain activity as bursts in the thalamic reticular nucleus (see Marlinski & Beloozerova, 2014; Sherman, 2001) and harmonic activity (e.g., the alpha waves originating from the occipital lobe; see Niedermeyer, 1997).

Another example is the activity change with the course of the circadian rhythm (see Welsh et al., 1995). These factors are known to determine the synchronization behavior of networks. However, their influence on spatiotemporal energy dissipation and the creation of DRNs may be limited because of the large-scale nature of network propagation considered here. Besides, our model did not incorporate the long- and short-range inhibitory mechanisms that are known to shape the spatiotemporal brain dynamics. These are doubtlessly essential factors in shaping brain dynamics. However, connectivity will remain the most critical one. We are looking forward to extended investigations of the functional expression of SC.

### Conclusion and Outlook

The whole-brain network perspective is very informative on a systems level. In its essence, it decomposes the brain into a system composed of nodes and links, capable of spatiotemporal pattern formation. This interpretation allows us to ask fundamental questions about stimulated pattern propagation, which is at the heart of information processing in the brain. By systematically stimulating all brain areas in the model, we have mostly mapped out all the network signal propagation pathways supported by the mouse brain model. It is remarkable that in the absence of any extensive parameter fitting, our model demonstrates a moderate, but robust, predictive power regarding signal propagation after stimulation of the sensory pathways.

We applied the catalog of DRNs (Figure 2) to the sensory pathways. Here, the predicted simulated responses to stimulation within a sensory modality showed consistency along the paths and were in line with the literature. Finally, we modified the model to meet the characteristics of the optogenetic stimulation in the in vivo experiments and compared in silico and in vivo brain responses. Our model could moderately replicate the realistic sequences of activations in the neocortex, even without parameter tuning (Figure 3). It suggests that mesoscale SC is an essential contributor in signal propagation in the neocortical networks.

In this study, using an in silico model, we individually applied focal stimulation, that is, separately to single brain areas. This approach allows sampling of the behavior outside of the attractor landscape and gaining more detailed knowledge of the network’s organization. An extension of this scheme and a better understanding of the role of the DRNs would be possible through the simultaneous stimulation of multiple brain sites. Unfortunately, to date, achieving such a complete picture is impossible even in silico due to a large number of possible combinations. However, we can extend the notion of the DRNs by stimulation of structures in two or multiple sensory pathways. Coordinating stimulation in such an approach would also contribute to a theoretical understanding of multisensory integration (Keil & Senkowski, 2018).

Interestingly, the default mode network arose in 11 out of 12 characteristic brain responses (DRNs) in the model, emphasizing the functional role of this network in the brain model. However, our presented results neither support the default mode network as a driver nor as epiphenomenal. To clarify the nature of the default mode network, in a potentially upcoming study, we could simultaneously stimulate two locations in the brain model. In that way, we can investigate the order of occurrence and interactions in brain activity and compare it to the catalog of individual stimulation responses. Another possibility is to use a more complex but realistic resting-state dynamic model (e.g., Hansen et al., 2015) and investigate the effect of stimulation.

In conclusion, we believe that our model provides a powerful tool for future studies, in which new hypotheses could be built and tested empirically. Our model provides mechanistic insight underwriting the fundamental nature of brain dynamics with until now unknown physiological significance.

## MATERIALS AND METHODS

To perform the exploration of brain dynamics in silico, we constructed a large-scale model for the mouse brain, consisting of the geometry, the SC, and the local dynamics. We performed simulations of the whole-brain network model in response to focal brain stimulation, decomposed the brain responses, and extracted the DRNs. We tested the predictability of the DRNs by the SC using appropriate statistical tools and compared previously known experimentally functional networks with the DRNs. We furthermore investigated the integration of brain stimulation and response along sensory pathways into the whole-brain network. Finally, we compared the simulated brain response to focal stimulation and VSD imaging data obtained during focal optogenetic stimulation in the mouse brain. The following sections provide a detailed description of each step.

### Modeling the Mouse Brain

We isotropically sampled the Allen Brain Atlas (ABA; http://connectivity.brain-map.org) with a resolution of 50 μm (Melozzi et al., 2017), resulting in a total number of 3,300,706 voxels (1,653,785 voxels for left side), which encode the injection by its spatial location and connectivity among 512 brain areas. To perform simulations of the mouse brain model on *The Virtual Brain* platform (Sanz-Leon et al., 2013, 2015; http://thevirtualbrain.org/), we preprocessed the data as follows. We triangulated the smooth surface of the isocortex using a regular mesh, distributed across 84 cortical areas (Figure 1C). The mesh consisted of 27,554 triangles and 41,524 edges (88 μm is the length of the shortest edge, 190 *μ*m is the longest edge, with a mean edge length of 125 μm) between 13,972 vertices (Figure 1A and Figure 1B, and Supporting Information Figure S2). Each area of the isocortex contains between 69 and 667 nodes in the mesh (Table 1 and Supporting Information Tables S1 and S2) and, because the mesh is regular, the number of nodes indicates the size of an area in the ABA. In addition to the areas in the isocortex, the model includes 428 subisocortical areas. In contrast to the isocortical brain areas, we lumped each of these 428 subisocortical areas in the model to a point in physical space (i.e., centroid). The mouse brain model is a network of 14,400 nodes (13,972 vertices for the 84 spatially extended areas on the isocortical surface, and 428 nodes, each for a nonisocortical brain area). We performed the division of the reconstructed isocortical surface into areas (i.e., the parcellation) by

1.  extending the normal vectors of each vertex to about 200 *μ*m inside the volume of the isocortex,
2.  determining the five closest injection sites, and
3.  evaluating their classification to an area of the isocortex in the ABA.

We further corrected the isocortical parcellation for holes and isolated/mismatched vertices.

To connect nodes, we distinguish short-range structural connectivity (SC) from long-range SC (Figure 1D–1F). The short-range SC connects nodes within an area, and between areas, if they are spatially close from one another with a homogeneous and isotropic connection probability decreasing with distance (Braitenberg & Schüz, 1991, 1998) (Figure 1D–1F). The long-range SC links all the nodes of an area with those nodes of another area (Figure 1D and 1E), based on the ABA. The long-range SC is specific to the pair of brain areas and thus heterogeneous. The tracer experiments, compiled in the ABA, mostly relate the spatial diffusion of the tracer to the injection sites, mostly in the right brain. The connectivity is consequently left/right symmetric in the mouse model because we used a mirrored connectivity pattern in the left hemisphere. The ratio of the projection density provides the long-range SC weights to the injection site volume (Oh et al., 2014). For that reason, results are shown for one hemisphere, although we performed the model simulations using both left and right hemispheres as well as the interhemispheric connections. For a sanity check, the simulated activity was correlated between the same area in the left and right brain and confirmed the left/right symmetries. Neighboring areas can exchange information via the short-range SC within the cortex and via the white matter tract, that is, long-range SC (e.g., Area 2 with Areas 1 and 3 in Figure 1D).

Each vertex point is a network node holding a neural mass model connected to other nodes via the short- and long-range SCs. When we stimulated an area, all the nodes of this area are simultaneously and homogeneously activated. Then, the stimulation-induced activity in each node decays differently according to the activity in the surrounding nodes via short-range SC and remote nodes via long-range SC. The ability to drive the network does not depend on the number of nodes within an area. In the model, mean activity across all nodes in a source area is transferred to all nodes in a target area via long-range SC.

We considered the ratio of short-range SC to long-range SC as a degree of freedom and performed parameter exploration (see Jirsa & Kelso, 2000; Qubbaj & Jirsa, 2007, 2009 for systematic studies with two-point connection). Others have previously estimated this ratio. For instance, Braitenberg and Schüz (1998) assessed that pyramidal cells have synapses in equal shares from long-range and local axons. The ratio of short- to long-range SCs mainly depends on

1.  the resolution of the used geometrical model of the cortex,
2.  the representation of the SC, and
3.  the network node description (e.g., canonical model, neural mass model).

The latter can incorporate local connectivity (see, e.g., Spiegler & Jirsa, 2013, for more detail). At the two extreme connectivity configurations, we expect two different behaviors.

1.  Two disconnected cerebral hemispheres with locally but homogeneously connected nodes by 0% of long-range SC (and 100% of short-range SC) allows activity to propagate only locally from a cortical stimulation site.
2.  The purely heterogeneously connected 512 brain areas with locally unconnected nodes by 100% of long-range SC (and 0% of short-range SC) allows activity to travel only long distances via white matter fiber tracts with time delays.

Furthermore, since the characteristic distance of short-range SC is less known (Spiegler & Jirsa, 2013), we also considered it as a parameter varying between 500 μm and 1,000 μm (Braitenberg & Schüz, 1998). We then systematically stimulated each of the 512 areas with a broad range of parameter values (for the ratio and the spatial range), resulting in a total of all 18,432 simulation trials.

Brain dynamics at rest have been found to operate near-criticality (Ghosh et al., 2008; Deco et al., 2011, 2013; Spiegler et al., 2016). Near-criticality is a state of a system that is on the brink of a qualitative change in its behavior (Shew & Plenz, 2012). The proximity to criticality predicts that the brain’s response to stimulation will primarily arise from structures and networks closest to instability. Activities in those networks require the longest time to settle into equilibria after stimulation and are associated with large-scale dependencies and scale invariance (Haken, 1978). The behavior is consistent with the center manifold theorem. It states that a high-dimensional system in a subcritical state will converge on a lower dimensional manifold (here few networks) when we stimulate a system.

Consequently, we equally set each node in the brain network model to operate close to its critical point, where the network shows no activity without stimulation. We used the stable regimen of each network node (i.e., stable focus) to stimulate a given area in the direction of its instability point (i.e., supercritical Andronov–Hopf bifurcation) and induce characteristic energy dissipation through the brain network. The short-range SC and long-range SC, as well as the associated signal transmission delays, and the local dynamics at the network nodes, constrained the dissipation of energy. In the network model, the operating point of every node disconnected from the network is at the same distance from its critical point, that is, the supercritical Andronov–Hopf bifurcation (Supporting Information Figure S1A). If the system reached the critical point, the node enters into a constant oscillatory mode. The SC determines the working distance to the critical point at each node in the network, at each timestamp by weighting and delaying the incoming activity from other nodes in the network. Hence, network metrics of the SC, such as the in-strength, that is, the sum of weights of incoming ties to a node, may indicate the distance of a node’s operating point to its critical point and thus criticality (Kunze et al., 2016). The network model, however, was set so that the system never reaches criticality. We achieved the subcritical behavior by normalizing the SC so that the maximum in-strength is one. It ensures that the mouse brain network segregates and integrates information while the activity decays in the network. That is because the network cannot amplify activity by SC less than one.

When we applied stimulation, the node operates closer to the critical point, and the response is in the form of a damped oscillation (Supporting Information Figure S1A). The closer a node operates to the critical point, the stronger the amplitude and the longer the decay time of the node’s response (Supporting Information Figure S1A). Since the nodes were working near-criticality, the response to the stimulation was transient and lasting a few milliseconds. The damped oscillation generated in one stimulated node is then sent via its efferent connections to its target nodes, triggering there, in turn, a damped oscillation (Supporting Information Figure S1B). If the network would mainly consist of nodes connected in series, the activity would decay very fast after the stimulation (Supporting Information Figure S1B). However, since the outgoing activity of a node can influence the nodes projecting back to it, recurrent systems appear (Supporting Information Figure S1C and S1D), which allow the activity to dissipate on a much longer timescale. After the initial decay, the evoked activity persists in the DRNs (Figure 2C and 2D), which may reflect feedback loops and reentry points in the SC. A DRN acts on perturbations due to, for instance, sensory stimuli or random fluctuations in the network (flexibility), and outlasts the stimulation (criticality).

Supporting Information Figures S2 to S5, as well as Table 2 and Table 3, display the described network properties. The differences in the responses stem from the proximity to criticality in brain areas. The proximity depends on the SC (in particular, the extent of recurrent networks) comprising the synaptic weights and the time delays (Figure 1). This behavior is predicted by the center manifold theorem, which is the mathematical basis for criticality (Haken, 1978).

The directionality of the long-range SC, derived from the tracer studies provided by the ABA, is assessed by two measures estimating the symmetry in the connectivity weight matrix. The first measure, *Q*_0_ = [0, 1], gives 0 if symmetric and 1 if asymmetric and reads:

$$Q_{0}=∥C-C^{\text{T}}∥/∥C+C^{\text{T}}∥,$$ (1)

where A is here the square matrix containing connection weights between the 512 areas, and ∥*C*∥ is the matrix norm. The second measure, *Q*_1_ = [0, 1] is defined as follows:

$$Q_{1}=∥C-C^{\text{T}}∥/∥2C∥,$$ (2)

and gives *Q*_1_ = 0 if the matrix C is symmetric and *Q*_1_ = 1 if antisymmetric. Both measures indicate a high degree of symmetry in the weight matrix of the ABA-SC and, therefore, a dominant bidirectionality of connectivity.

### Large-Scale Brain Model

Dynamics of a vector field *Ψ*(*x*, *t*) at time *t* ∈^1^ and position *x* ∈^3^ in space Ω are described by a delay-integro-differential equation:

$$\begin{array}{c} \partial_{t}\Psi (x,t)=F\left(\Psi (x,t)\right)-a_{I}I(x,t)\\ \;+(1-\alpha )a_{L}\int_{L}\text{d}X^{\prime }\Psi (x-X^{\prime },t)g(X^{\prime })+\\ \;+\alpha a_{\Omega }\int_{\Omega }\text{d}X^{\prime }\Psi \left(x-X^{\prime },t-\left|x-X^{\prime }\right|/v\right)H(X)C\left(∥x-X^{\prime }∥/v\right)K^{T}(X^{\prime }), \end{array}$$ (3)

where ∂_*t*_ is the derivative with respect to time, *t*. The input *I*(*x*, *t*) allows the stimulation dynamics to intervene on a node. The operator *F*(Ψ(*x*, *t*)) locally links variables of the vector field. The scalar *α* balances short- and long-range SCs (first and second integral) in the vector field. The vectors, *a*_*I*_, *a*_*L*_, and *a*_Ω_ of factors relate the input *I*, and both types of SC to the vector field Ψ(*x*, *t*). The kernel *g* (*x*) describes the short-range SC. The field is time delayed due to a finite transmission speed *v* via the long-range SC given by matrix *C*(*x*). The vectors *H*(*x*) and *K*(*x*) establish the links among the long-range SC and the targets and the sources. Note that the transmission speed enters the second integral concerning long-range SC. We assumed the transmission via the short-range SC (first integral) to be instantaneous (to reduce the computational expenses) to perform the parameter exploration study. We describe the spatial and temporal aspects of the model in more detail in the following two subsections.

### Geometry and Structural Connectivity

The spatial domain Ω = {*L*_1_ ∪ *L*_2_ ∪ *S*} separates both cerebral hemispheres *L* = {*L*_1_ ∪ *L*_2_}: left, *L*_1_ and right, *L*_2_, from subisocortical areas *S*, that is, ⋂Ω = ∅. Two open surfaces describe the geometry of the left and right isocortex (*L*_1_ and *L*_2_) by a regular mesh of 27,554 triangles with 41,524 edges between 13,972 vertices. The short-range SC follows a Gaussian distribution *g*(*x*) = exp(#x2212;*x*^2^/(2*σ*^2^)), which is invariant under translations on *L* (Spiegler & Jirsa, 2013). Each open surface, *L*_1_ and *L*_2_, is divided into *m* = 42 areas, that is, *L*_1_ = ∪_*r*∈*R*_1__*A*_*r*_ and *L*_2_ = ∪_*r*∈*R*_2__*A*_*r*_ with *R*_1_ = *R*(*m*), *R*_2_ = *R*_1_ + *n*: *R*(*λ* ∈) = {*r* | *r* ∈, *r* ≤ *λ*}, where *n* = 428 is the number of subcortical areas. The division of the surfaces into areas is based on the ABA, *A*_*r*_ = *A*(*r* ∈) ∈ Ω: →^3^ onto space Ω for introducing long-range SC. We lumped each of the *n* = 428 considered subisocortical areas to a single point in space *S* = ⋃_*r*∈*R*_3__*A*_*r*_ with *R*_3_ = *R*(*n*) + 2*m*. The long-range SC links all the vertices of an area with the vertices of another area. Note that every 428 nonisocortical areas represent a node in the large-scale brain network. Connections between these nodes are point-to-point connections. Connections between an area of the isocortex and one of the 428 nonisocortical areas are links between all nodes inside the isocortical area and the node representing the nonisocortical area. The long-range connections, *C* transmit mean activities of sources to target areas, *H*(*x*) and *K*(*X*′) with a finite transmission speed, *v* = 1 ms^−1^. The square matrix, *C*(∥*x* − *X*′∥/*v*) contains (2*m* + *n*)^2^ weights, *c*_*ij*_(∥*x* − *X*′∥/*v*): *i*, *j* = 1, …, 2*m* + *n* taken from the ABA. The row vectors *H*(*x*), and *K*(*X*′) contain 2*m* + *n* operations, *h*_*i*_(*x*), and *k*_*j*_(*X*′) on the targets and sources, respectively. The operations are *h*_*i*_(*x*) = *δ*_*x*_(*A*_*i*_) and *k*_*j*_(*X*′) = *δ*_*X*′_(*A*_*i*_)/|*A*_*j*_| with the Dirac measure *δ*_Ω_(*A*) on Ω and the cardinality |*A*_*r*_| of the set *A*_*r*_.

The large-scale brain network model (Equation 1) is fully compatible with previous TVB descriptions (Sanz-Leon et al., 2015; Spiegler & Jirsa, 2013; Spiegler et al., 2016). Note that we used the set notation here to describe brain areas. We described the division of the SCs on both isocortices in the cerebral areas with the same notation.

### Local Dynamics

The vector field describes a two-dimensional flow (Stefanescu & Jirsa, 2008) linking two variables Ψ(*x*, *t*) = (*ψ*_1_*ψ*_2_)^*T*^(*x*, *t*) in (1) as follows:

$$F\left(\Psi (x,t)\right)=\eta \left(\begin{array}{c} \psi_{2}(x,t)-\gamma \psi_{1}(x,t)-\psi_{1}^{3}(x,t)\\ -\varepsilon \psi_{1}(x,t) \end{array}\right).$$ (4)

The number of 28,800 variables thus represents the network state of the mouse brain model. The parameterization *γ* = 1.21 and *ε* = 12.3083 sets an isolated brain area close to a critical point, that is, an Andronov–Hopf bifurcation (sketched in Supporting Information Figure S1) with a natural frequency around 42 Hz using a characteristic rate of *η* = 76.74 *s*^1^. This rhythm in the gamma band accounts for the local activity, such as a coordinated interaction of excitation and inhibition (Buzsáki & Wang, 2012). It is not explicitly modeled here but represented by the surrogate parameter *γ*. This damping factor *γ* determines the local excitability (i.e., the critical distance to the Andronov–Hopf bifurcation) and is thus a surrogate for the ratio of excitation and inhibition at each location of the brain network. The Dirac delta function is applied to a brain area, *I*_*r*_(*x*, *t*) = −5*ηδ*_*x*_(*A*_*r*_)*δ*(*t*). The connectivities and the input act on the first variable *ψ*_1_(*x*, *t*) in (1) by *a*_*L*_ = *a*_Ω_ = (*a*_*I*_)^*T*^ = (*η* 0). The connectivity-weighted input determines criticality by working against inherent energy dissipation (i.e., stable focus) toward the bifurcation. So as to avoid passing the bifurcation, both short- and long-range SCs, *g*(*x*) and *C*(∥*x* − *X*′∥/*v*), are normalized to have unity maximum in-strength across time delays by (i) d*x* g(*x*) = 1 and (ii) $\sup_{\lambda \in \Omega /v}\left\{\sum_{j}^{2m+n}c_{ij}\left(∥\lambda ∥\right)\right\}$ = 1.

### Simulation

We simulated the whole-brain mouse model on a computer using the large-scale brain network simulation engine The Virtual Brain (TVB). We discretized the model concerning physical space and time, to run simulations on a digital computer. The mesh of 7,020 nodes (and 6,952 nodes) evenly filled the surfaces of the left isocortex *L*_1_ (and the right isocortex *L*_2_). Subcortical structures in *S* remain unaffected by the discretization. The geometry of the brain is captured in physical space, Ω, by a net number of 14,400 nodes (i.e., 13,972 cortical and 428 subisocortical nodes). We rewrote the spatial integrals in (1) in terms of matrix operations, where the long-range SC remains the same. For that matrix version of the short-range SC, we spatially sampled the short-range SC on the cerebral surfaces (Spiegler & Jirsa, 2013). We used Heun’s method to solve the resulting system of difference equations with a time step of 40 *μ*s for 1 second per realization. Each realization had a unique parameter setting. We sampled the following parameters: 512 stimulation sites, SC balance α = {0.0, 0.2, 0.4, 0.6, 0.8, 1.0}, and characteristic short-range SC distance, σ/μm ∈: 500 σ/μm 1,000. The spatial cutoff range for short-range SC was eight times the standard deviation of the connectivity kernel 8σ (Spiegler & Jirsa, 2013). That gives the six configurations of the short-range SC. We verified the implementation by the algebraic solution of an isolated node (i.e., no connections) and by the field properties (e.g., compact solutions spreading radially around a stimulation site) of the homogeneously linked cerebral nodes.

The lower bound of the spatial range of σ = 500 μm results from the used geometrical model for the isocortex. A nearly regular mesh of triangles approximates each cerebral hemisphere with a finite edge length of 125 μm on average (see Supporting Information Figure S2). The cortical meshes on the isocortices spatially sampled the continuous Gaussian kernel for the short-range SC. Because of the finite edge lengths in the mesh, the characteristic distance of the short-range SC should not fall below 207.78 μm for −3 dB cutoff of spatial frequencies concerning their magnitude (Spiegler & Jirsa, 2013). The lower bound of the spatial range of σ = 500 μm for the homogeneous Gaussian connectivity kernel causes a loss of at least 4.55% of spatial information (mainly short-range), which corresponds to −17.37178 dB cutoff (see Spiegler & Jirsa, 2013).

### Stimulation and Decomposition

We uniformly stimulated all network nodes of a brain area for a period of the characteristic time of the nodes, that is, *η*^−1^, to evoke damped oscillations with a maximum of one. We calculated the stimulation-induced activity in the brain responses by subtracting the stimulation response of an isolated node from the response of stimulated nodes in the network. Principal component analysis (PCA) was performed on the induced brain responses by using the covariance matrix among the 14,400 network nodes’ time series. The time series were selected in the time interval of 250 to 750 ms after the stimulus onset (similar to Spiegler et al., 2016). For further analysis, we considered up to three principal components that hold more than 99% of the variance across conditions.

### Extracting Dynamically Responsive Networks

We extracted the DRNs by

1.  determining the induced component of generated brain activity after stimulation,
2.  decomposing the multivariate time series (of the 14,400 neural masses) of stimulation-induced activity (in the period between 250 and 750 ms after stimulation onset *t*_0_) into principal components (PCs: eigenvectors and corresponding eigenvalues),
3.  correlating the eigenspaces spanned by the eigenvectors that cover 99% of the variance in the brain activity (eigenvalues),
4.  clustering the eigenspaces and
5.  rotating the eigenspaces onto a common basis, and
6.  lumping the clusters.

The decomposed activity patterns after focal stimulation resulted in step 2 are called ss-DRNs. We identified a total of 18,432 ss-DRNs for all stimulation sites and all connectivity parameter configurations. In contrast to the ss-DRNs, the DRNs are accessible from several locations in the brain by stimulation. Similar to ss-DRNs, DRNs are composed of three components. The components result from aligning and averaging correlated PCs of ss-DRNs within each cluster (steps 3 to 6).

The dot product of the corresponding normalized eigenvectors (i.e., PCs), obtained from the PCA of the stimulation responses (i.e., ss-DRNs), was used to measure the similarity of the dissipation across different stimulation sites for a range of values of the balance of the SC and a spatial range of the short-range SC. We clustered the eigenspaces using a *k*-means algorithm based on the similarity measure for each SC balance and each range of the short-range SC. We estimated the number of clusters by using the gap statistic (Tibshirani et al., 2001). For each cluster, we rotated and aligned the eigenspaces onto the basis. The cluster basis is the eigenspace with the highest similarity among all cluster members. That was achieved using the singular value decomposition and calculating the optimal rotation matrix (Kabsch, 1978). Averaging the aligned basis vectors (i.e., PCs) of eigenspaces in a cluster gives the set of eigenvectors for each cluster. The resulting vector indicates the contribution of each node (e.g., belonging to a cortical or a subcortical structure) to a DRN.

### Statistics on the DRNs and Prediction of the DRNs by the Structural Connectivity

To perform statistics on the catalog of DRNs, we extracted and correlated the DRNs from the 14,400 time series. We pairwise correlated each of the three components of each of the 24 DRNs, that is, 12 DRNs by stimulation of left areas and 12 DRNs by stimulation of right areas (comprising 72 components composing the 24 DRNs). The significance level of 1% was Bonferroni corrected by the total number of components. In addition, three thresholds were applied to the correlation values *Corr* in order to find strong (*Corr* > 0.8), good (0.6 < *Corr* 0.8), and moderate agreements (0.5 < *Corr* 0.6) among the 72 components of the 24 DRNs. The analysis revealed a total of five significantly distinct motifs in the DRNs, indicating links between DRNs.

To test to what extent the topology in the structural connectivity predicts the brain activity in response to stimulation, we calculated the following graph-theoretic measures on the long-range SC: in-degree (number of afferent connections), out-degree (number of efferent connections), total-degree, in-strength (afferent projections and arriving activity), out-strength (efferent projections and exiting activity), total-strength (the combination of both), and clustering coefficient (Rubinov & Sporns, 2010). We measured incoming, outgoing, or all connected ties to an area regarding (1) their numbers and (2) their weights. By counting the connections, we obtained the in-, the out-, and the total-degree. By calculating the sum of connection weights, we obtained the in-, the out-, and the total-strength. The clustering coefficient measures the degree to which areas in a graph tend to group. We correlated the activity (after stimulation) with the graph-theoretical characterization across network nodes.

Furthermore, we correlated the activity at the network nodes after stimulation with the connectivity strengths of the direct afferent (incoming connection to an area) and efferent connections (outgoing connection from an area). We compared each of the nine measures, applied to the brain areas in the long-range SC, with the components of each DRN (i.e., the eigenvector). We used the Pearson correlation, the rank correlation (Kendall’s tau), and the Bhattacharyya coefficient for the correlation. We used the same permutation test to test statistical significance as for the comparison of the DRNs with the functional networks. We Bonferroni corrected the significance level of 5%.

### Motifs Connecting DRNs

The pairwise correlation of the three components of the DRNs revealed five consistent activity patterns that recur in the components of several DRNs. Because of the recurrence, we call them motifs. Furthermore, to clarify the role of the five motifs in dealing with stimuli, the motifs were correlated with experimentally known functional networks.

### Functional Networks in the DRNs

Based on the ontology of ABA, we mapped the cartographic description of the resting-state networks onto the mouse brain’s structural model to determine whether networks that are dynamically responsive to stimulation (DRNs) resemble the experimentally known spatial activity patterns. We followed the description of the networks by Sforazzini et al. (2014) and by Zingg et al. (2014). In our geometrical model, the resultant map describes the probability of contribution of an area to a particular functional network by the following three contribution levels according to the references (Sforazzini et al., 2014; Zingg et al., 2014):

1.  no contribution for unmentioned,
2.  medium contribution for mentioned, or
3.  high contribution for explicitly emphasized.

We then used the Pearson correlation and the Bhattacharyya coefficient (Bhattacharyya, 1946) to estimate the amount of overlap (i.e., the square root of the inner product) between a functional network and a motif of the DRNs. The averaged eigenvectors within a cluster are the components of each DRN. The components of each eigenvector were squared and summed within each area to obtain coarse-grained eigenvectors. We normalized the resultant eigenvectors to unit length. Then, we compared functional networks and each normalized coarse-grained component of a DRN. We corrected the *p* values due to 27 multiple independent comparisons (nine functional networks with three eigenvectors per stimulation site) for a significance level of 5%.

### Sensory Pathways

If not explicitly cited, the primary reference for the description of the sensory pathways of the mouse brain is the book authored by Watson, Paxinos, and Puelles (2011). The visual pathways run from the retina through the hypothalamus, midbrain, and thalamus to terminate in the primary visual cortices. The retinal ganglion cells bilaterally connect to the hypothalamus. Moreover, all cells project to the (sensory related) SuCo, according to the ipsilateral visual hemifield. The (sensory-related) SuCo projects, through the pulvinar nucleus and lateral posterior nucleus of the thalamus, to the areas of the cerebral cortex that are involved in controlling eye movements (Stein & Meredith, 1991). Pulvinar nuclei are virtually nonexistent in the rat and grouped as the lateral posterior-pulvinar complex with the thalamic LPN due to its small size in cats. In humans, it makes up roughly 40% of the thalamus, making it the largest thalamic nucleus (LaBerge, 1999).

Furthermore, the SuCo also receives activity from other sensory systems such as whisker movements (Stein & Meredith, 1991). The retinal ganglion cells have ramifications to contralateral structures in the midbrain, namely OT, OPT, and PPT (Pak et al., 1987). The retinal ganglion cells also have ramifications that run into the V1 through the thalamus, namely IGL, vLGN, and dLGN, according to the ipsilateral visual hemifield (abbreviations of brain areas are given in Supporting Information Tables S1 and S2). Intergeniculate IGL seems to play an essential role in mediating phases of the circadian rhythms that are light insensitive, as well as phase shifts that are light dependent (Harrington, 1997; Edelstein & Amir, 1999). Supporting Information Figure S7A summarizes the network with specific visual hemifield connections.

The auditory pathways originate from the cochleae to the A1 through the medulla (DC and VC), the pons (NLL), the midbrain (IC and BIC), and the thalamus (MG). Supporting Information Figure S7B summarizes the specific contra- and ipsilateral connections.

The trigeminal nerve connects the whiskers with the somatosensory areas in the isocortex. The nerve runs through the medulla, the pons, the midbrain, and the thalamus to finally terminate in the barrel fields of the somatosensory areas. In the different brain structures, each whisker is represented in a discrete anatomical unit, called a barrel (see Supporting Information Figure S7C). Parts of the medulla, the pons, the thalamus, and the isocortex show topographic organization of the major facial whiskers. Supporting Information Table S2 lists names and abbreviations of nuclei. The somatotopy of whisker follicles in S1 and S2 mainly comes via the thalamus. In the thalamus, the VPM shows a somatotopic arrangement of barreloids fed by the trigeminal nerve nuclei through the pons (PrV) and the medulla. In the medulla, the SpVc shows a somatotopic arrangement of barrelets. Specifically, the trigeminal nerve fibers have ramifications to SpVc, SpVi, and SpVo (medulla), to the PrV (pons), and the MeV (midbrain; Bosman et al., 2011). Via the MeV, whisker movements can activate the sensory-related parts of the superior colliculus of the midbrain (Matesz, 1981; Ndiaye et al., 2000). The SuCo has other parts related to sensory processing, such as the visual system and eye movements. The pathway via PrV and VPL to the S1 conveys primarily somatotopic information of whiskers. The other minor pathways are as follows: the PrV (part of the pons) mainly projects to the dorsomedial part (and less to the head area) of the VPM and less to the Po (both thalamus). The Po receives connections from the ventral posteromedial nucleus, the SpVi, and SpVo and sends input to the S1, S2, and M1 (Bosman et al., 2011; Lane et al., 2008). The SpVc sends input to the VPM and the LD. Both send input to the somatosensory areas. Supporting Information Figure S7D summarizes the specific contra- and ipsilateral connections.

The pathways from the fore and hind limbs pass via dorsal root ganglion to the ipsilateral dorsal column nuclei of the medulla, comprising the CN and the GN. The upper limb, and especially the forelimb, connects through the CN, whereas the lower limb, and especially the hind limb, connects through the GN. The nuclei of the medulla connect to the contralateral VPL (thalamus) via the medial lemniscus. The VPL has ipsilateral ramifications into the S1. Supporting Information Figure S7E summarizes the specific contra- and ipsilateral connections.

We have quantified the quality of the sensory pathways represented in the ABA connectome. Let us consider a particular set of structural connections (SCs) embedded in a large-scale brain network forming subnetworks, which are involved in the processing of a particular brain function. Such a large-scale brain network contains directed connections, whose weights are assumed to be positively-defined, that is, between network nodes *i* and *j*: *c*_*ij*_ ≠ *c*_*ji*_, and *c*_*ij*_ ≥ 0 (the first index is the target, and the latter is the source). A particular network comprises a set **R** of edges aka connections **R** ⊆ **r**_*n*_ between index vertices *i*∈ and *j*∈ aka nodes in physical space Ω, **r**_*l*_ = {*j* → *i*} with *i*, *j* = 1, …, 2*m* + *n*. The arrow in the notation for the *l*∈-connection **r**_*l*_ indicates a directed projection from vertex *j* onto vertex *i*. Then, we can characterize the embedding of a given connection by its particular in- and out-strength in a given large-scale brain network or by the maximum in- and output value

$$W(r_{l})\left\{\begin{array}{c} \frac{c_{ij}}{\sum_{b}c_{ib}}\;\text{or}\;\frac{c_{ij}}{\max_{b}(c_{ib})}\;\text{for}\;\text{target}\;i\\ \frac{c_{ij}}{\sum_{a}c_{aj}}\;\text{or}\;\frac{c_{ij}}{\max_{a}(c_{aj})}\;\text{for}\;\text{target}\;j \end{array}\right.,$$ (5)

where the maximum income of a target *i* is max_*b*_(*c*_*ib*_) and the maximum output of a source *j* is max_*a*_(*c*_*aj*_). Using both graph-theoretic measures color the edges r_*l*_ with a positive scalar *q*_*l*_

$$q_{l}(r)=c_{ij}^{2}\times \left\{\begin{array}{c} \frac{1}{\sum_{a}c_{aj}\sum_{b}c_{ib}}<1\;\text{colored by in- and out-strength measure}\\ \frac{1}{\max_{a}(c_{aj})\max_{b}(c_{ib})}\leq 1\;\text{colored by maximum input and output} \end{array}\right.$$ (6)

We quantified the presence of a subnetwork of interest that is defined by *N* connections R ⊆ r_*l*_: *n* = 1, …, *N*, in a large-scale network U: R ⊆ U as follows:

$$R/U=\frac{1}{N}\overset{N}{\underset{l=1}{\sum }}q_{l}(r).$$ (7)

We used both normalization types to quantify the embedding of sensory pathways.

To study the response to stimulation of the pathways, we characterized activity patterns in terms of (1) the most active structures (and most active areas) as well as (2) the most active brain areas (irrespective of the total activity of structures), lateralization and symmetry.

Because the statistics on the DRNs (see section Statistics on DRNs in the Result section) indicate that the responses are best predicted (although weak) by the strengths of the directly outgoing connections from the stimulation site, we analyzed the spatial spread of the energy in the brain responses (integral of the power over time, i.e., .2 s to .8 s after stimulation). To test whether the activity is focal or spread over the brain (or within a structure), we determined the brain areas that contain more than 99% of the total energy in a brain response (entire brain and within a brain structure). Brain areas were then ranked to their strength of activity, and compared between left structures and contrasted with the homolog on the right. To clarify whether activity spreads laterally, we determined the brain areas that contain more than 99% of the total energy in the responses of the entire brain model and compared the location (left/right). Activation is symmetric to the stimulation site when the response to stimulation of a particular structure in one hemisphere is homologous with the stimulation response to stimulation of the same structure in the opposite hemisphere. The activation patterns are tested on bilateral symmetry by correlating the activity in the left areas with the activity at homolog areas on the right. A bilateral symmetric response means that the activity in the left side’s brain areas is similar to the ones on the right. An antisymmetric bilateral response means that the activity on the left side is the reverse of that on the right. Insignificant correlations (significance level of 5%) do not support either type of symmetry.

So far, we have analyzed the brain response to the stimulation of brain areas in each sensory pathway independently. To clarify whether the brain responses are consistent for a specific sensory pathway summarized in Supporting Information Figure S7, for example, the auditory pathways from the cochlea to the primary auditory areas, we used the catalog of DRNs as described in the section Statistics on DRNs in the Results.

### Observer System for VSD Imaging

The observer system is a physical forward solution that links the state system (the mean postsynaptic potential and current) for the 2D model at each network location to an experimentally observable measure. To compare the simulated and empirical activity in the isocortex in response to stimulation, we projected the simulated brain activity onto the plane that reconstructs the focal plane from which the camera setup captures the VSD images (Mohajerani et al., 2013). Since the VSD imaging signal shows high correlations with the low-frequency component of local field potentials (Mohajerani et al., 2013), the VSD imaging signal possibly reflects postsynaptic potentials of neural populations. Therefore, we considered a direct link between the local dynamic model in the mouse brain network and the VSD imaging. To obtain smooth nonoscillatory responses in simulated VSD signals, the Hilbert transform of the oscillatory signals, generated by the state-state system, was used. We performed that preprocessing step because the VSD imaging optical signal is not typically oscillatory (Mohajerani et al., 2013).

The VSD imaging provides a spatially flattened image of the brain activity while the surface of the isocortex in physical space is curved. To find a projection form the curved surface of isocortex, considered in our network model, to the flat focal plane of the VSD imaging, we used the coordinates of the primary sensory areas in the model and the imaging data. We found these coordinates in the VSD imaging data by using peripheral stimulation (Mohajerani et al., 2013). We used these coordinates to find a projection plane (size given by the camera sensor used for the VSD imaging) in the model that best corresponds to the VSD imaging data (see Figure 4). This mapping, then, provides a direct link between the local postsynaptic potential in the model and the measured VSD images. The isocortical areas, captured by the VSD imaging setup (see, for more details, Mohajerani et al., 2013), are listed in Table 5. The simulated and measured activity can thus be compared on the pixel level of the images as well as on the coarse-grained level of brain areas, using the region mapping.

### Comparing Voltage-Sensitive Dye Imaging and Simulated Data

VSD imaging measures the intensity of emitted light from the superficial layers of the isocortex stained with VSD molecules. The light intensity, normalized by its baseline, reflects the neuronal membrane potential. Thus, we separately calculated the ratio of the deviation of the baseline optical signal to the baseline (Δ*F*/*F*_0_) per pixel. Then we converted this ratio to the percentage by multiplying it by 100. We defined the baseline as the prestimulus level of the optical signal for each pixel. A signal level of 0.2% in a particular pixel means that the optical signal captured a 0.2% deviation from baseline. We then used the Δ*F*/*F*_0_ signal to identify the activated pixels at each time point after the stimulation was applied. We defined the onset of activation for a pixel as the time stamp, at which the pixel activity surpasses 20% of its peak activity. We obtained the temporal order of activation among these regions by calculating the onset of the activation for all the isocortical regions in our imaging window.

Moreover, we calculated the maximum of the Δ*F*/*F*_0_ signal for each pixel around a temporal neighborhood of the stimulation timestamp. We obtained the maximum intensity maps presented in Figure 3F and Supporting Information Figures S8 and S9. These maps ease the qualitative investigation of the overlap of activated regions in the simulated and empirical data.

For comparing model and in vivo data, we used the following exemplary model configuration: 500-μm short-range SC with a ratio of 40% short-range SC to 60% long-range SC (i.e., *α* = 0.4), and the stimulation period of *η*^−1^ = 13 ms. The size of the stimulation focus was adjusted to mimic the laser stimulation with a beam diameter of 360 *μ*m on the surface in the center of isocortical areas.

We used the following six steps to calculate the similarity between two spatiotemporal patterns of brain activity evoked by the stimulation in vivo and in silico.

1.  We calculated the sequence of activated regions ordered from earliest to latest for each spatiotemporal pattern of activity (suppose the two sequences are ABCDEF and ACHBFG, for example).
2.  We excluded the first region in each sequence (we get BCDEF and CHBFG).
3.  A subsequence starting with the first element and with a specific length was chosen from each sequence (if the chosen length is 4, we get BCDE and CHBF).
4.  The corresponding nonoverlapping elements of the two subsequences were made identical (leading to BCDE and CDBE). After this step, we made the two subsequences from the same elements.
5.  We calculated Kendall’s tau distance (Kendall, 1938) between the two subsequences. This distance is the same as the number of transpositions (swapping of adjacent elements) to convert one subsequence into another. The value of this distance is then divided by (*n* − 1) *n*/2, which is the maximum number of transpositions between two sequences with length *n* (the distance between BCDE and CDBE is then 0.33).
6.  We defined the unnormalized similarity between the two subsequences as one minus the value obtained in step 5 (unnormalized similarity between BCDE and CDBE is 1 − 0.33 = 0.67).
7.  We multiplied the value obtained in step 6 with the ratio of the number of overlapping elements to the total number of elements in each subsequence to get the similarity value between the two subsequences (similarity between BCDE and CDBE is 0.67/2 = 0.335).

We used the median of similarity values between only in vivo stimulus-induced spatiotemporal patterns of activity as a benchmark. We then evaluated the meaningfulness of similarity values.

### In Vivo VSD Imaging and Optogenetic Stimulation in The Mouse Brain

We used adult ChR2 transgenic mice (line 18, stock 007612, strain B6.Cg-Tg (Thy1-COP4/EYFP) 18Gfng/J; The Jackson Laboratory) for in vivo imaging experiments. These transgenic mice express the light-activated ion channel, Channelrhodopsin-2 (ChR2), in layer 5B pyramidal neurons across the cortex under the control of the mouse thymus cell antigen 1 (Thy1) promoter. We housed the animals in clear plastic cages in groups of two to five, under a 12:12 h light/dark cycle. Mice were given ad libitum access to water and standard laboratory mouse diet at all times. The University of Lethbridge Animal Care Committee approved the animal protocols following the guidelines set forth by the Canadian Council for Animal Care. To perform the VSD imaging experiments, we anesthetized the animals with 0.5% isoflurane. Then, we performed a 7 × 6 mm unilateral craniotomy (bregma 2.5 to 4.5 mm, lateral 0 to 6 mm). We removed the underlying dura as described previously (Kyweriga et al., 2017). We incubated the exposed brain for 60 minutes with the dye RH-1691 (Optical Imaging, New York, NY) solution prepared with the HEPES-buffered saline (0.5 mg/ml). Thirty minutes after we washed out the unbounded dye solution from the brain, we covered the stained brain surface with the 1.5% agarose dissolved in HEPES-buffered saline and sealed with a glass coverslip. We removed the underlying dura. Throughout surgery and imaging, body temperature was maintained at 37°C degrees using a heating pad with a feedback thermistor. The VSD data was collected as 12-bit images using a CCD camera (1M60 Pantera, Dalsa, Waterloo, ON) and EPIX E4DB frame grabber with XCAP 3.8 imaging software (EPIX, Inc., Buffalo Grove, IL). We set the focal plane to about 1mm below the cortical surface to reduce the potential brain surface pulsation artifacts. VSD was excited with the red light emitted from a LED (Luxeon K2, 627-nm center) and filtered in the range of (630 ± 15) nm. We filtered the VSD fluorescence using a 673–703-nm optical bandpass filter (Semrock, New York, NY) before being captured by the CCD camera. To correct for time-dependent changes in VSD signals that accompany all imaging, we also collected several nonstimulation trials that we used for normalization of stimulated data. We used a 10-s interval between each stimulation (optical or sensory). To reduce potential VSD signal distortion caused by the presence of large vessels, we focused on the cortex to a depth of ∼1 mm. In our previous work, we measured VSD fluorescence across the cortex by using histology and demonstrated relatively high labeling at even ∼750 *μ*m in-depth (Mohajerani et al., 2010; Greenberg et al., 2018). Nevertheless, to reduce regional bias in the VSD fluorescence signal caused by brain curvature or due to uneven brain staining, all the optical responses were expressed as a percent change relative to baseline (Δ*F*/*F*_0_ × 100%). The total duration of excitation in a typical imaging experiment ranged from 1,200 to 1,500 s.

We identify the coordinates of the sensory areas by performing periphery stimulation combined with VSD imaging. We performed the peripheral somatosensory stimulation by delivering 0.2 to 0.5 mA, 1-ms electrical current to either forelimb or hindlimb. The visual stimulation was a 1-ms pulse of combined blue and green light to the contralateral (left) eye. For the auditory stimulation, we played a sound clique next to the left ear of the mouse. We performed tactile stimulation of a single whisker (C2) by using a piezoelectric device (Q220-A4-203YB, Piezo Systems, Inc., Woburn, MA). We used the output of a diode-pumped solid-state laser emitting light with a wavelength of 473 nm (Shanghai Dream Lasers Technology, ShangHai, China) to stimulate ChR2-expressing neurons. The laser beam was directed to defined points of stimulation (grids; 750 *μ*m) on the cortical surface using Ephus toolbox (Suter et al., 2010). That Matlab (Mathworks, Natick, MA) toolbox controlled the galvanometer scan mirrors (Cambridge Tech, Lexington, MA), via analog output voltage from PCI-6110 DAQ (National Instruments, Austin, TX). The Ephus program controlled the overall timing of individual optical stimulation trials with TTL triggers to XCAP software. Low amplitude (7.5 to 20 mW/mm2) and short duration (1 ms) single collimated (f-number = 36) laser beam (120 μm) were applied to the cortical surface to ensure sufficient activation, low laser stimulus artifact, and low photobleaching. We stimulated each site 5 to 10 times (10-s interstimulus interval) and averaged replicate responses.

## ACKNOWLEDGMENTS

We thank Eunice Hoyee Chan for proofreading of the manuscript.

## SUPPORTING INFORMATION

Supporting Information for this article is available at https://doi.org/10.1162/netn_a_00152, http://connectivity.brain-map.org (Oh et al., 2014), http://www.mouseimaging.ca/research/mouse_atlas.html (Dorr et al., 2008), www.MouseConnectome.org (Hintiryan et al., 2016), and http://thevirtualbrain.org/ (Sanz-Leon et al., 2013).

## AUTHOR CONTRIBUTIONS

Andreas Spiegler: Conceptualization; Formal analysis; Investigation; Methodology; Project administration; Software; Validation; Visualization; Writing - Original Draft; Writing - Review & Editing. Javad Karimi Abadchi: Investigation; Validation; Visualization; Writing - Review & Editing. Majid Mohajerani: Investigation; Supervision; Validation; Writing - Review & Editing. Viktor K. Jirsa: Conceptualization; Formal analysis; Funding acquisition; Methodology; Supervision; Writing - Review & Editing.

## FUNDING INFORMATION

Viktor K. Jirsa, Fondation pour la Recherche Médicale (FRM), Award ID: DIC20161236442. Andreas Spiegler, European Commission’s Human Brain Project, Award ID: H2020- SGA1: 720270, SGA2: 785907, SGA3: 945539. Viktor K. Jirsa, French National Research Agency (ANR), Award ID: ANR-17-RHUS-0004, EPINOV. Viktor K. Jirsa, French Institute of Health and Medical Research (Inserm, International Laboratory Associated Program Epi-Surge) and the SATT Sud-Est, Award ID: 827-SA-16-UAM. Andreas Spiegler, Deutsche Forschungsgemeinschaft (http://dx.doi.org/10.13039/501100001659), Award ID: SFB936 Z3. Majid Mohajerani, Canadian Network for Research and Innovation in Machining Technology, Natural Sciences and Engineering Research Council of Canada (http://dx.doi.org/10.13039/501100002790), Award ID: 40352.

## TECHNICAL TERMS

Dynamics: Concerned with the study of the time evolution of a system’s state variables.
Structural connectivity (SC): The set of all material connections, typically comprising short-range and long-range axonal projections within and between brain regions.
Energy: The integral of power, i.e., the square of brain activity over time. Induced activity, and thus, energy dissipates after stimulation.
Trajectory: The path in state space that is traced out as the state variables evolve in time.
Dynamically responsive network (DRN): A coherent activation of brain regions to focal stimulation.
Motif: is a recurrent activation pattern in the brain following stimulation.
Voltage-sensitive dye (VSD): Changes its spectral properties with the membrane potentials. Optical imaging captures these changes as a proxy for neuronal electrical activity.
State variables: The quantities that unambiguously define the state of a system.
Viral vector tracer: A viral agent that causes a fluorophore expression along the axonal projections in the mouse brain.
Criticality: The set of characteristic behaviors that occur when a system approaches the critical point of a qualitative transition (bifurcation) from one behavior to another.
State space: The space spanned by the state variables.
Bifurcation: The mathematical mechanism describing the qualitative change of behavior of a system.
Emergence: The process of self-organization describing the formation of an ordered state of the system.
Optogenetic stimulation: Uses light to activate ion channels/pumps inserted in neuronal membranes through genetic modification.
