# Supplementary material for: In silico exploration of mouse brain dynamics by focal stimulation reflects the organization of functional networks and sensory processing
Source: Netw Neurosci. 2020 Sep 1;4(3):807–51. doi: 10.1162/netn_a_00152 (PMC7888484; doi:10.1162/netn_a_00152)
Supplement: Supplementary file 1 [file netn-04-807-s001.pdf]

Spiegler, A., Abadchi, J. K., Mohajerani, M. & Jirsa, V. K. (2020). Supporting information for "In-silico Exploration of Mouse Brain Dynamics by Focal Stimulation Reflects the Organization of Functional Networks and Sensory Processing." *Network Neuroscience*, *4*(3), 807-851. https://doi.org/10.1162/netn_a_00152

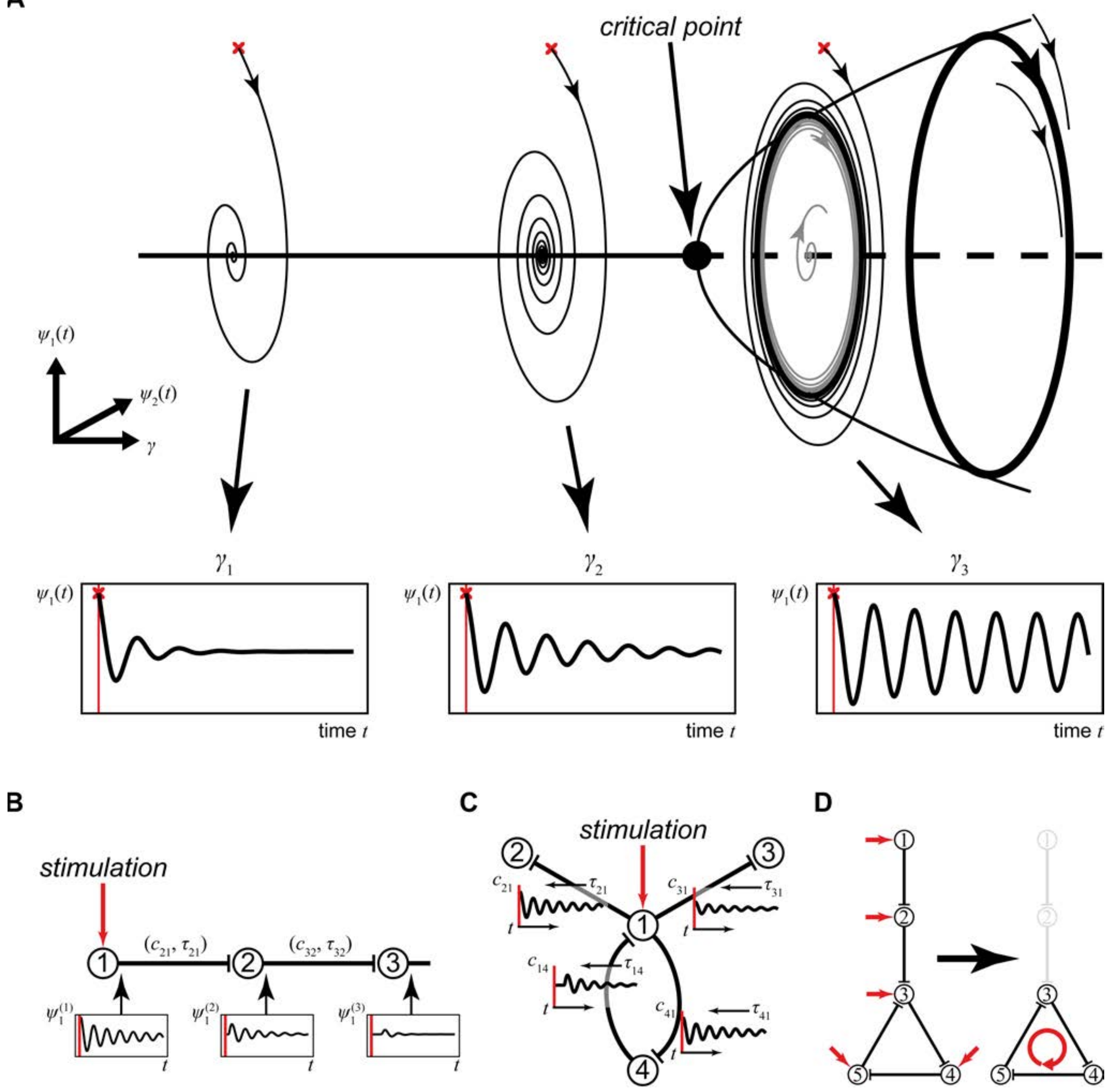

Supplementary Figure 1. The large-scale brain model works near criticality. Panel **A**: Each node in the network model is parameterized by γ to operate intrinsically at the same distance from the critical point if unconnected. A node shows oscillation (42 Hz) in response to stimulation (red crosses); otherwise, the activity is zero in the absence of stimulation. The activity at each node is determined by two time-dependent variables, $\psi_1(t)$ and $\psi_2(t)$. The closer a node operates to the critical point, the larger and the longer lasting the oscillation becomes (compare γ_1 and γ_2). When the critical point is reached, the node intrinsically performs a rhythm of constant magnitude. The model, however, is set so that the critical point is never exceeded. Panel **B**: Principles of activity spreading after stimulation. The damped oscillation generated in the stimulated node (1) is transmitted via its efferent connections to its target node (2), triggering there, in turn, a damped oscillation with weaker amplitude and faster decay, which then propagates to the next node. Activity $\psi^{(j)}{}_1(t)$ of the node (j) is scaled by c_{ij}, delayed by τ_{ij} transmitted to the node (i) via short- and long-range structural connections (SCs). In such a chain, the activity would decay fast. Panel **C**: In the large-scale brain model, multiple activity re-entry points can be found. At any time point, the dynamics of a node is influenced by all incoming activity. The response of the node to stimulation (1) is relayed to linked nodes (2–4), which may be fed back to 1 via 4 and may allow the induced activity to dissipate on a much longer time scale. The network response thus depends upon the SC, which allows the network to operate near criticality. Panel **D**: Activation of the DRNs. Activity after stimulating a node (1 or 2) in a series connection decays fast (as in panel **B**). However, activity may circulate and thus decays slower in a feedback network (of nodes 3–5). Such remaining activity after the initial stimulation decay reveals the DRNs.

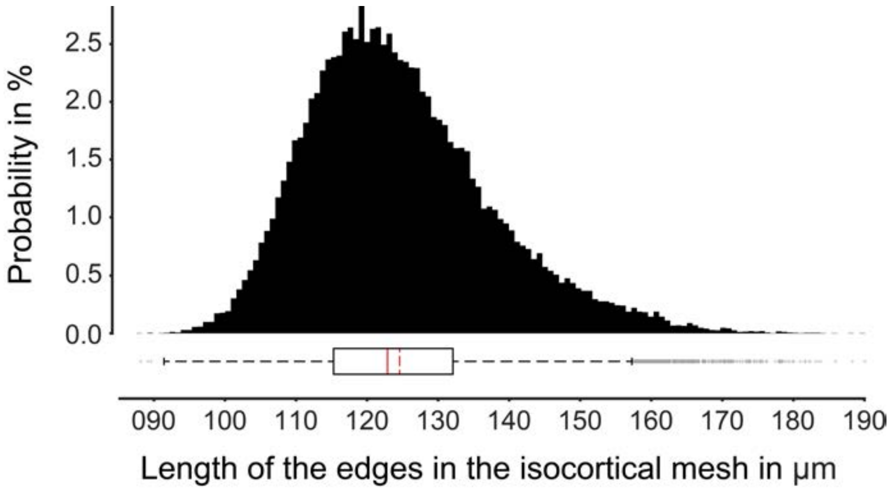

Supplementary Figure 2. The sharp unimodal distribution of edge lengths indicates the quality of the reconstructed surface of the isocortex, using a regular-triangle mesh of 27,554 triangles with 41,524 edges between 13,972 vertices. The expectation and the standard deviation is (124.5786 ± 13.0144) µm. The edge lengths are positively skewed (0.7164) due to the positivity of length (minimum and maximum lengths are 88.1525 µm and 190.0629 µm).

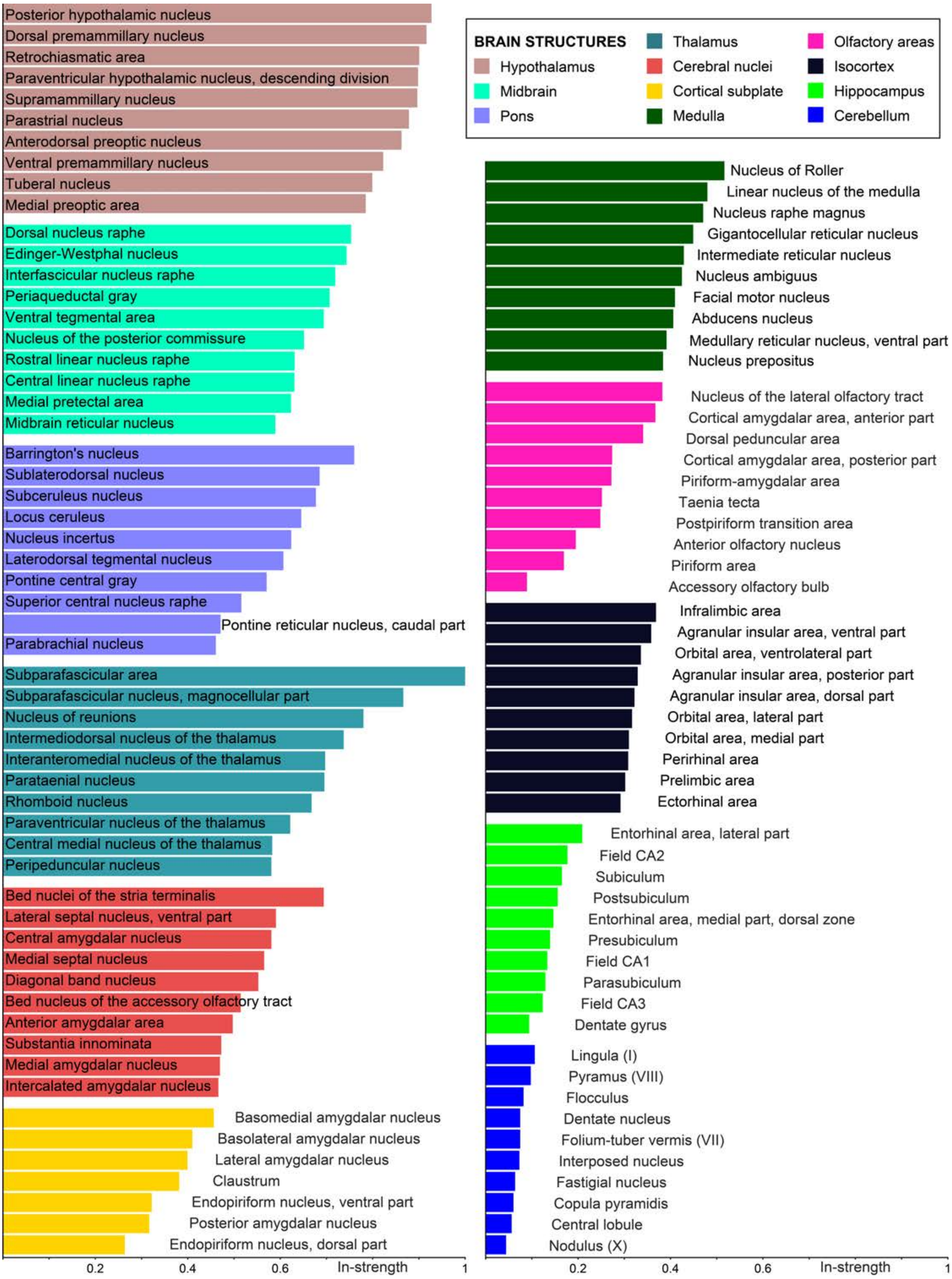

Supplementary Figure 3. In-strength indicates the incoming projections to a brain area. The hypothalamus receives the strongest input in the mouse brain model and the cerebellum the weakest. The isocortex is listed 9 out of 11 structures. The structures are ordered by their mean in-strength. This indicates that the subcortical structures are highly interdependent and the isocortex might be sensitive to subcortical input while it can be autonomous as well. For each structure (e.g., thalamus), 10 areas are listed in order of the highest in-strength. Note that the cortical subplate is divided in seven areas.

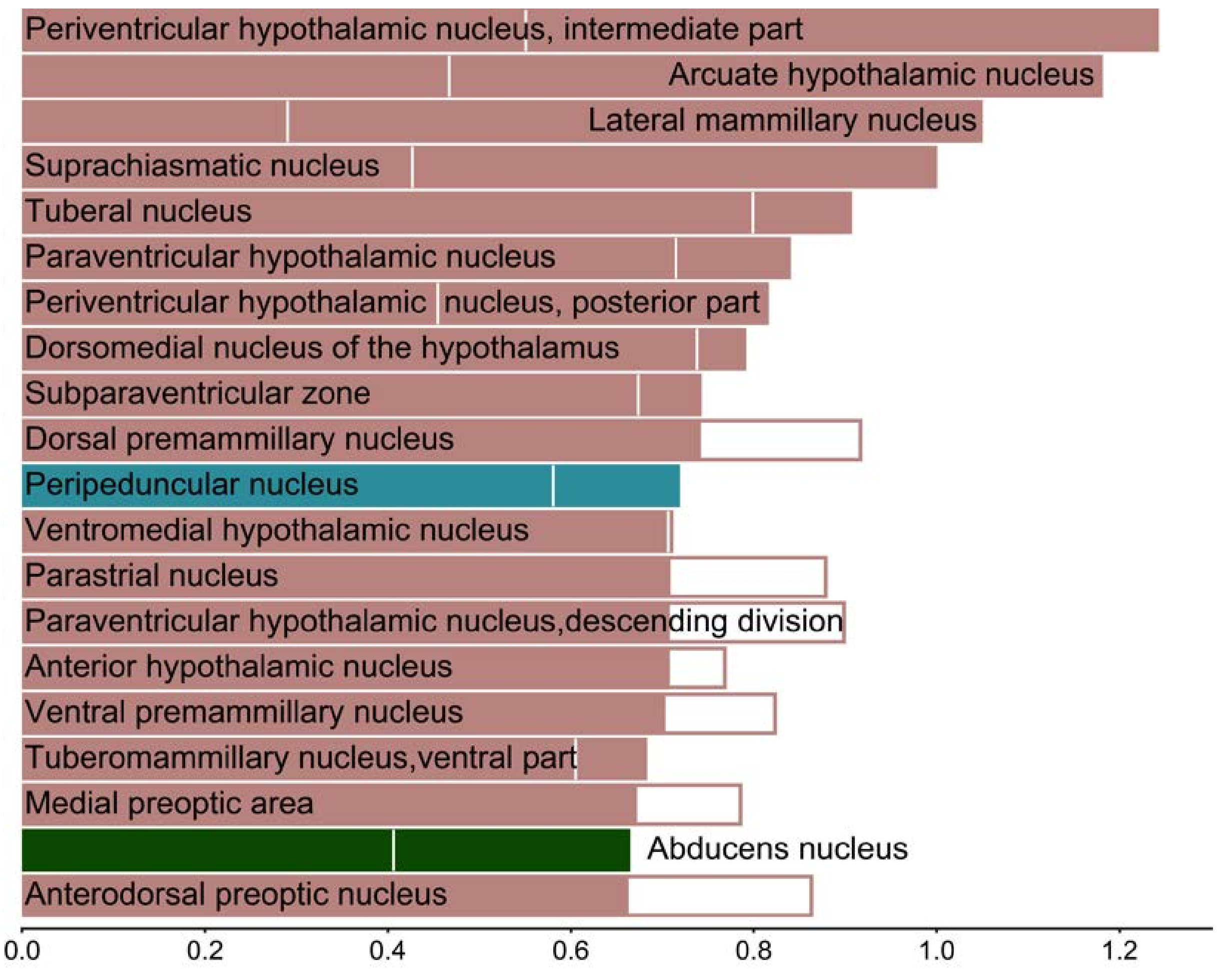

Supplementary Figure 4. Out-strength indicates that, in the mouse brain model, areas in the hypothalamus have the highest number of projections to downstream regions. The color code for the structures is the same as in Supplementary Figure 3: brown indicates areas in the hypothalamus, dark cyan the thalamus, and dark green medulla. The white vertical lines in a bar and the white filled bars indicate the in-strength of an area.

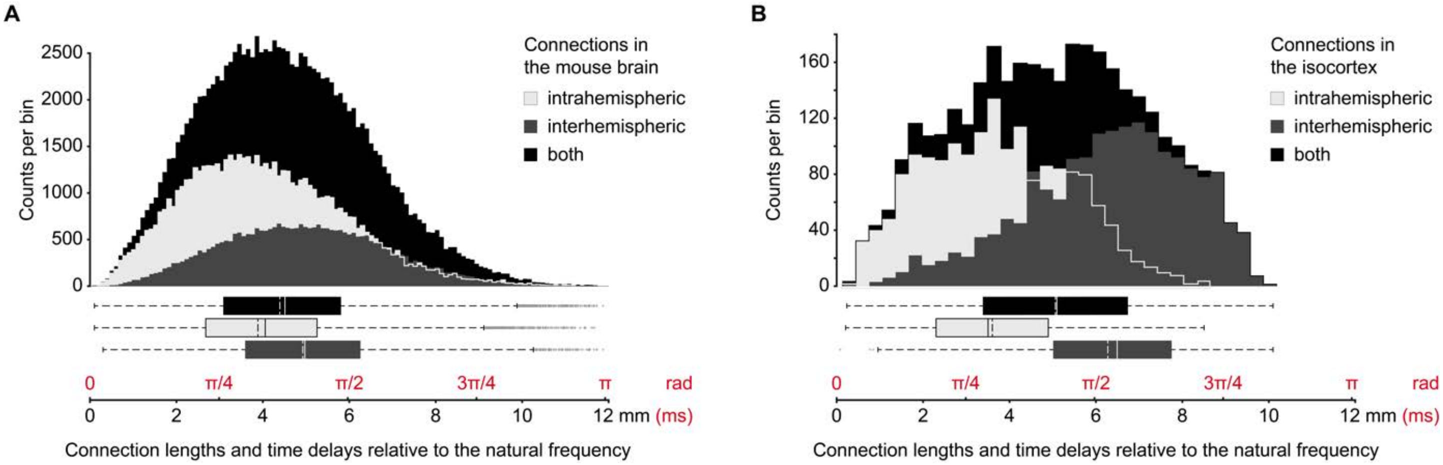

Supplementary Figure 5. Histograms of the distances between brain areas from the ABA used to approximate the tract lengths of long-range connections. Panel **A**: The distribution of connections in the entire mouse brain is slightly skewed but unimodal with a mean and median of about 4.511 mm and 4.394 mm. The time delays in signal transmission shift the local activity by half a period via the most extended connections and mostly by about 3π/8 for medium-length connections. The distances directly translate into time delays by assuming an average conduction speed for white matter axonal fibers of 1 m/s. The connections in the entire mouse brain appear of similar length (panel **A**), while the connections only in the isocortex differ in length (panel **B)**. The intrahemispheric connections were shorter than the connections between the isocortical hemispheres. This result confirms the report in Braitenberg and Schüz, 1998, Chapter 26, of a second peak in the histogram whose significance is obscured. The isocortical connections were outnumbered, thus their quantitative effect on the entire mouse, in panel **A**, is marginal. Time delays translate into shifts of transmitted local brain activity. The local activity in the network model of the mouse brain is assumed to primarily convey the natural frequency at each brain area (about 42 Hz). The time delays translate into a phase shift in local activity throughout its transmission. The number of bins is 119 with a bin width of 0.0889 mm in panel **A** and 17 bins of 0.3056 mm width in panel **B**. The number of bins in the histogram, n_{bins}, was calculated according to $n_{\text{bins}} = \exp(0.626 + 0.4 \log(n - 1))$, with the number of connections n (Otnes and Enochson, 1972), which is listed in Table 2. Table 3 provides the statistics.

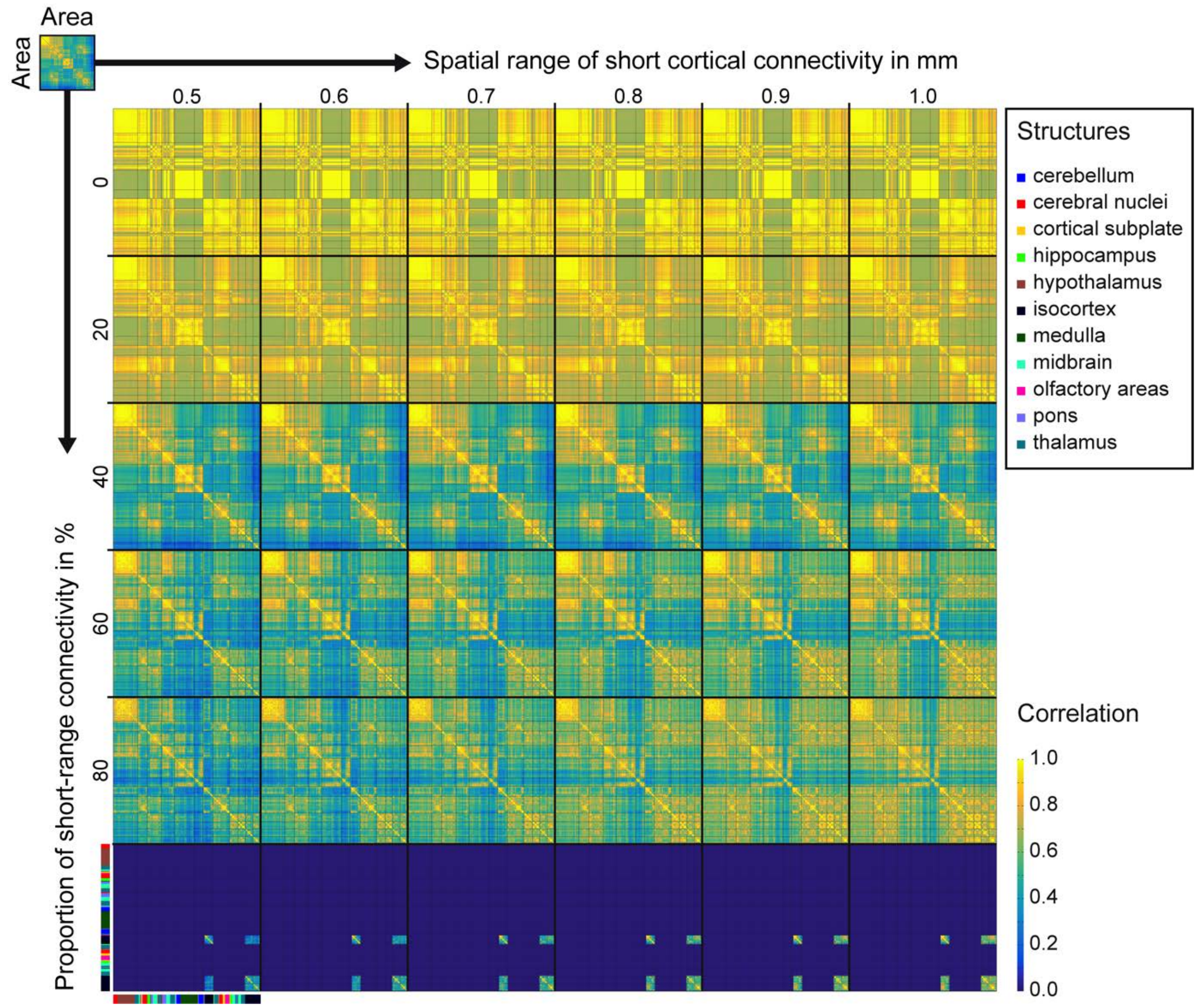

Supplementary Figure 6. Similarity matrices of the ss-DRNs reveal a consistent formation of spatially similar activity in the mouse brain. Each of the 512 brain areas is stimulated individually, and the 512 ss-DRNs are compared with each other resulting in one similarity matrix per connectivity parameter configuration. That is summarized in a similarity matrix for a given proportion of homogeneous short-range to heterogeneous long-range SCs (varied along rows) and for a given length of short-range connections (varied along the columns). The similarity of ss-DRNs increases with warmer colors (yellow indicates the maximum correlation of two ss-DRNs). The stimulation sites (areas) are equally sorted for all similarity matrices. The color bar on the axes of the lower-left matrix (500 µm spatial range and 100% of short-range SC) displays the structures to which a brain area belongs (e.g., nucleus accumbens and fundus of the striatum are cerebral nuclei in the mouse model). Though the results indicate clusters of DRNs, the organization of the similarity matrices was almost unchanged concerning the length of short-range SCs (similarity matrices along the columns). Long-range SC (see the row of 0% short-range SC) supported ss-DRNs that differ from those supported by the short-range SC (see the row of 100% short-range SC). However, both similarity matrices merge in the mix of short- and long-range SCs (see the similarity matrices along the rows).

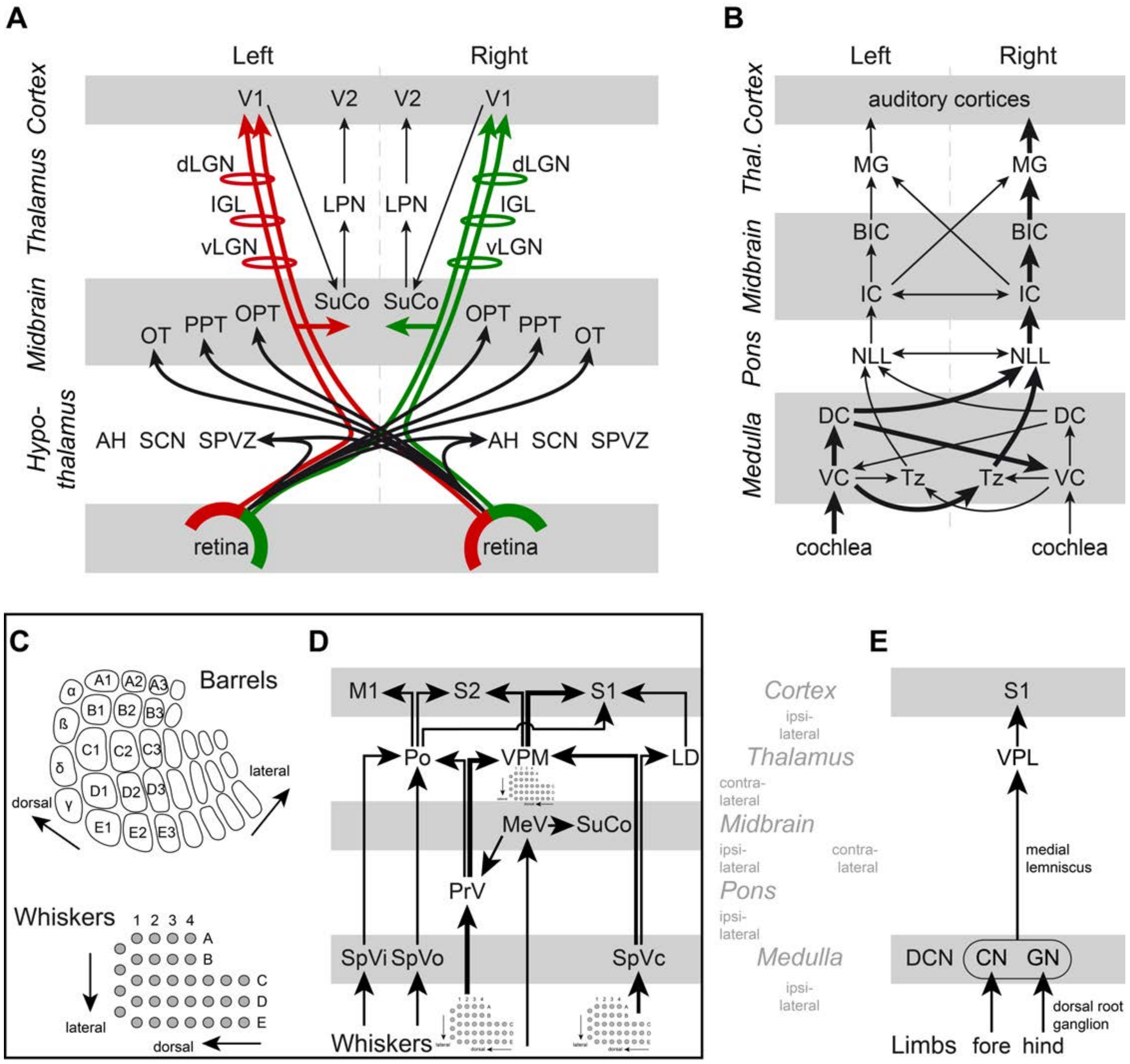

Supplementary Figure 7. Networks of sensory pathways. Panel **A**: The visual pathways run from the retina through the hypothalamus, midbrain, and thalamus and terminate in the primary visual cortices. The retinal ganglion cells bilaterally connect to the hypothalamus, and all cells project to the SuCo according to the ipsilateral visual field (colored links). The SuCo projects through the LPN of the thalamus to the areas of the cerebral cortex that are involved in controlling eye movements. Note that the SuCo also receives projections from areas controlling whisker movements. The retinal ganglion cells have ramifications to contralateral structures in the midbrain (OT, PPT, OPT) and ramifications that run into the primary visual cortex through the thalamus (vLGN, IGL, dLGN) according to the ipsilateral visual field. The projections from the visual fields through the retinal ganglion cells are highlighted in panel **A** by red and green colored links. The arrowheads indicate the termination areas of the axonal fibers. Panel **B**: The auditory pathways originate from the cochleae and run through the medulla, pons, midbrain, and thalamus to the primary auditory cortices. The thickness of the links in panel **B** indicates the strength of connectivity. Panels **C**–**D**: The pathways from the whiskers to the barrel fields run through the medulla, pons, midbrain, and thalamus and terminate in the isocortex. Panel **C**: The somatotopic map of the whiskers where each individual whisker is represented in a discrete anatomical unit, called a barrel. The map shows the correspondence of the topographical organization of the major facial

whiskers through the trigeminal nerve in the nuclei in the medulla (SpVc), the pons (barrelets), and through the thalamus (barreloids in VPM) to the somatosensory cortices (barrel cortex). Panel **D**: The pathways from the facial whiskers to the isocortex. The somatotopy of the whisker follicles mainly comes to the somatosensory cortices (i.e., S1, S2) via the trigeminal nerve nuclei (PrV in the pons and SpVc in the medulla) and the thalamus (VPM). The thickness of the links in panel **D** indicates the strength of connectivity. Whisker movements activate the SuCo in the midbrain through the MeV. The SuCo is also related to the visual system (see panel **A**) and, in particular, to eye movements. Panel **E**: The pathways from the fore and hind limbs pass via dorsal root ganglion to the ipsilateral dorsal column nuclei (DCN) in the medulla (CN and GN). The upper limb, and especially the forelimb, connects through the CN, whereas the lower limb, and especially the hind limb, connects through the GN in the medulla. The nuclei of the medulla connect via the medial lemniscus to the contralateral thalamus (VPL), which has ipsilateral ramifications into the primary somatosensory cortex (S1). The sensory networks in panels **A**, **B**, **D**, **E** are based on textbook descriptions (e.g., Watson et al., 2011) and include the relevant structures given by the ABA. The sensory pathways can be described using the ABA. However, the ABA needs refinements to distinguish lower and upper limb (especially hind and forelimb), see panel **E**. The ABA does not include a division of the barrel field of the primary somatosensory areas (compare panels **C–D)**. The networks in panels **A**, **B**, **D**, **E** indicate information flows to areas that do not necessarily terminate in the primary sensory areas of the isocortex, such as the hypothalamic and midbrain targets of the retinal ganglion cells in panel **A,** and the midbrain nuclei related to whisker movements in panel **D**. These well-known connections are usually not discussed regarding sensory processing in textbooks. As a result, visual and whisker system meet in the SuCo regarding eye and whisker movements. Note that the model is agnostic about the (sensory) information, meaning that nuclei may be activated, but the object of processing (information) is less defined. Meaning is attached by the vast amount of studies about the physiology of the nuclei. For instance, the hypothalamic nuclei play a role in the circadian timing system, and the nuclei in the midbrain (OT, PPT, OPT) receive input from the retina (see panel **A**). They are known to be involved in eye movement coordination and reflexes. The ABA includes the following areas involved in sensory processing. Brain areas and abbreviations are listed in Supplementary Table 1 and Supplementary Table 2.

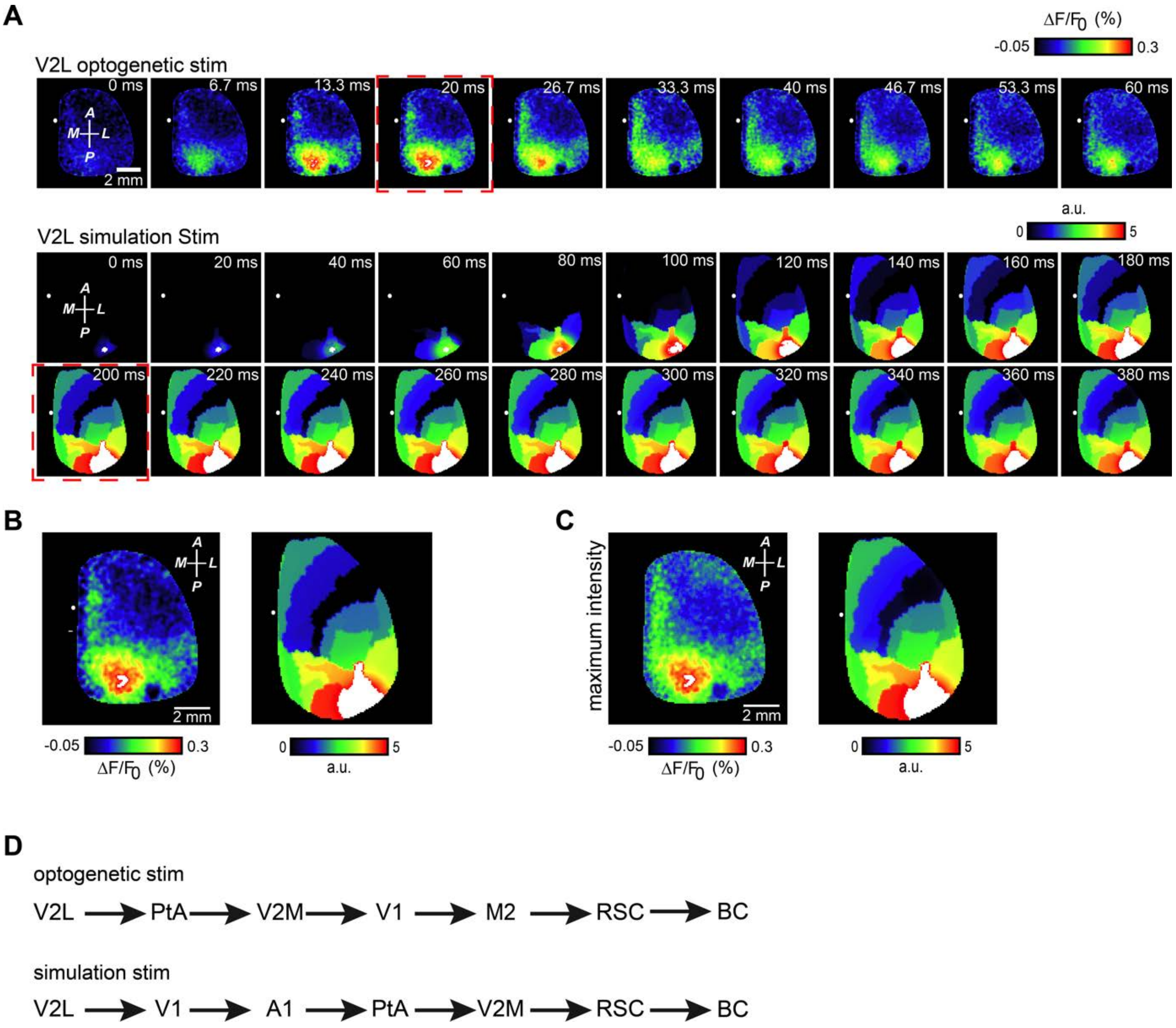

Supplementary Figure 8. Panel **A**: The spatiotemporal pattern of the voltage activity after the optogenetic (top) and the simulated (bottom) stimulation of the V2L. Panel **B**: The enlarged frames, specified by a red rectangle in Panel **A**, juxtaposed for qualitative comparison. Panel **C**: The spatial distribution of the post-stimulus maximum voltage activity. Panel **D**: The temporal order of activation of neocortical regions after optogenetic (top) and simulated (bottom) stimulation. Activation timestamp is the time at which the post-stimulus voltage level of a given area surpasses 20% of its peak activity.

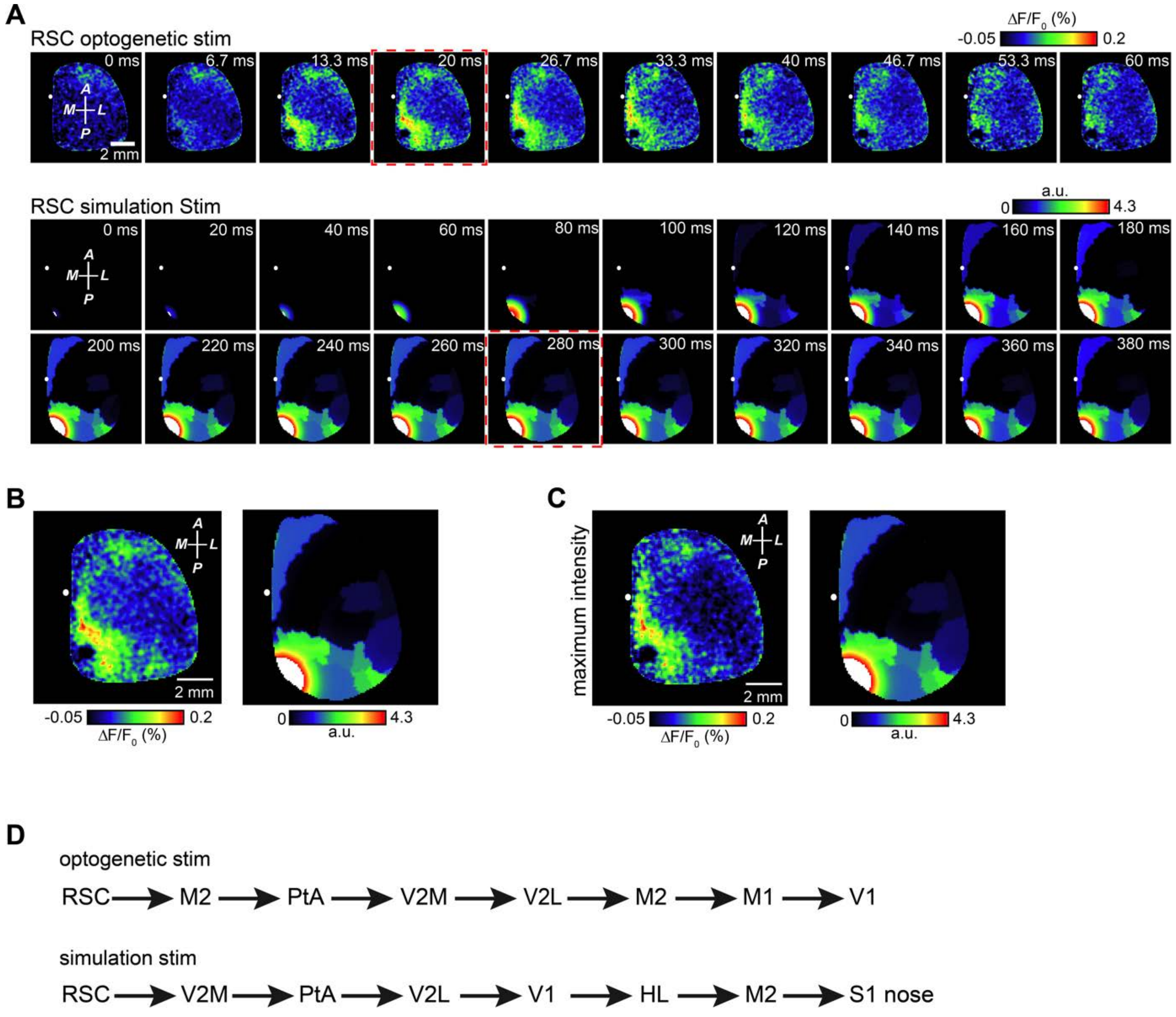

Supplementary Figure 9. Brain response after the optical stimulation of the retrosplenial cortex. Panel **A**: Spatiotemporal pattern of voltage activity after optogenetic (top) and simulated (bottom) stimulation of RSC. Panel **B**: The enlarged frames, highlighted by a red rectangle in Panel **A**, juxtaposed for qualitative comparison. Panel **C**: Spatial distribution of the post-stimulus maximum voltage activity Panel **D**: Temporal order of activation of neocortical regions after optogenetic (top) and simulated (bottom) stimulation. Activation timestamp is the time at which the post-stimulus voltage level of a given area surpasses 20% of its peak activity.

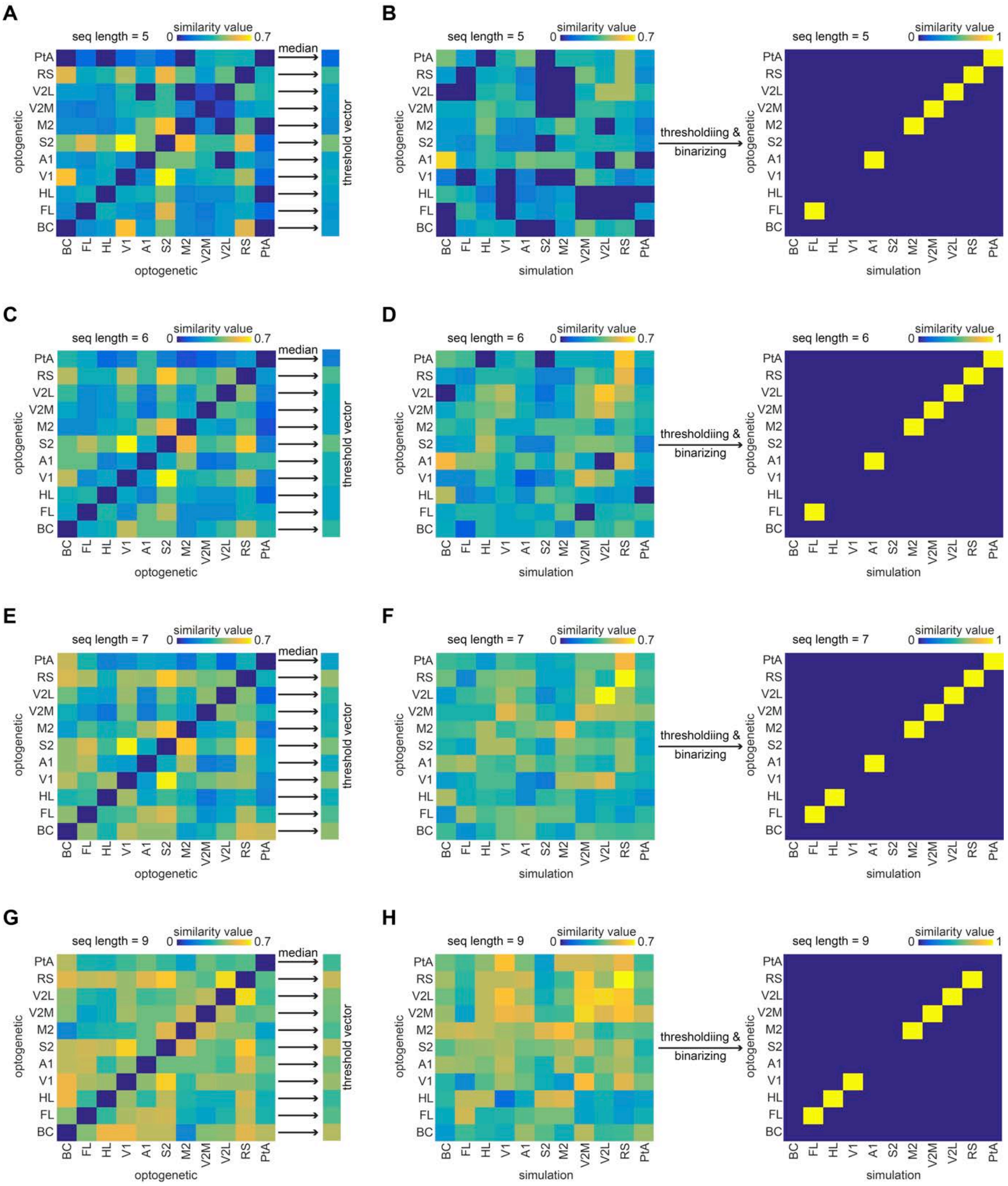

Supplementary Figure 10. The role of sequence length in similarity values between the empirical and the simulated stimulus-induced spatiotemporal pattern of activity in the neocortex. Panel **A**: The similarity matrix consisting of the similarity values between all the pairs of the temporally-ordered sequences of activated regions with length 5 induced by optogenetic stimulation of neocortical regions. The values on the diagonal of the matrix (self-similarity that is always 1) were purposefully removed. The median of each row gives an expected value for the similarity of the spatiotemporal patterns of activity induced by stimulation of the corresponding and rest of regions. Panel **B**: (Left) The same as panel **A** but for similarity values between empirical versus simulated spatiotemporal patterns of activity induced by focal stimulation of different neocortical regions. (Right) The similarity matrix between empirical and simulated patterns was thresholded and binarized using the threshold vector generated in Panel **A**. To highlight how similar the empirical and simulated patterns were, only the values on the diagonal are presented. Panels **C**-**D**, **E**-**F**, and **G**-**H** are the same as Panels **A**-**B** but for sequences lengths 6, 7, and 9, respectively.

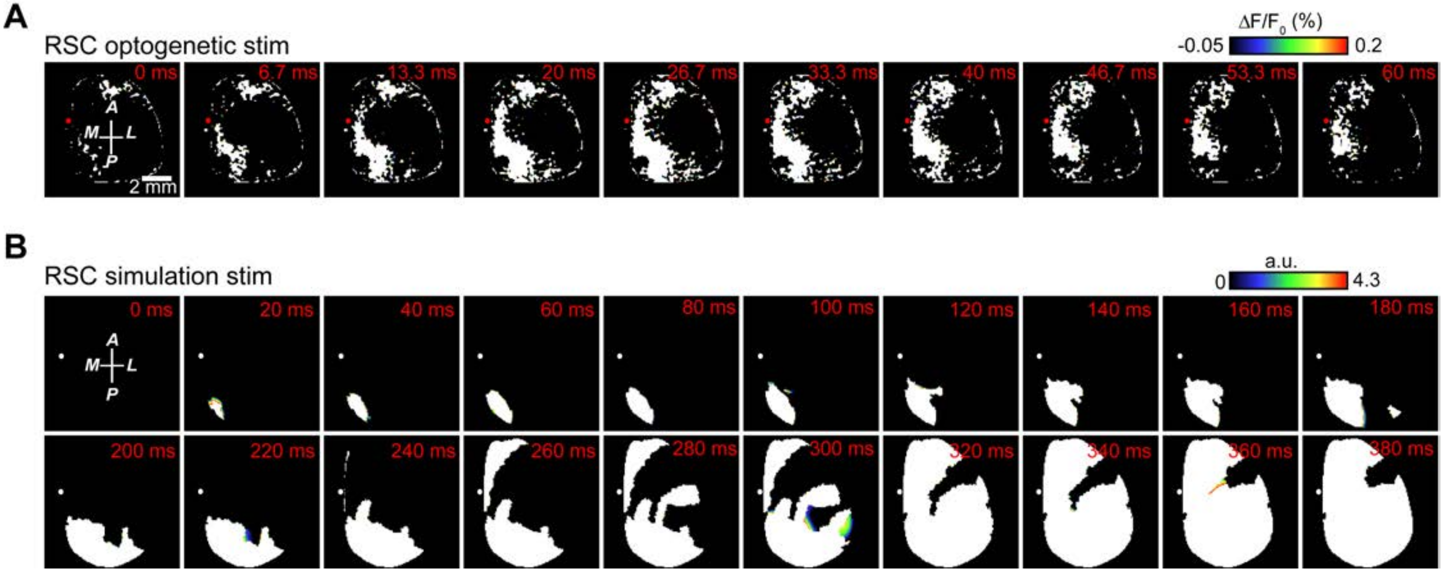

Supplementary Figure 11. Spatiotemporal pattern of activated cortical regions after optogenetic (**A**) and simulated (**B**) stimulation of RSC. A region is defined as activated when its activity surpasses 20 percent of its peak activity after stimulation occurred. (**A**) Schematics of the two axes of polarity AP and ML of RSC in (**A**) and evolution of activated cortical region (in white pixel) with time in (**A**) and (**B**).

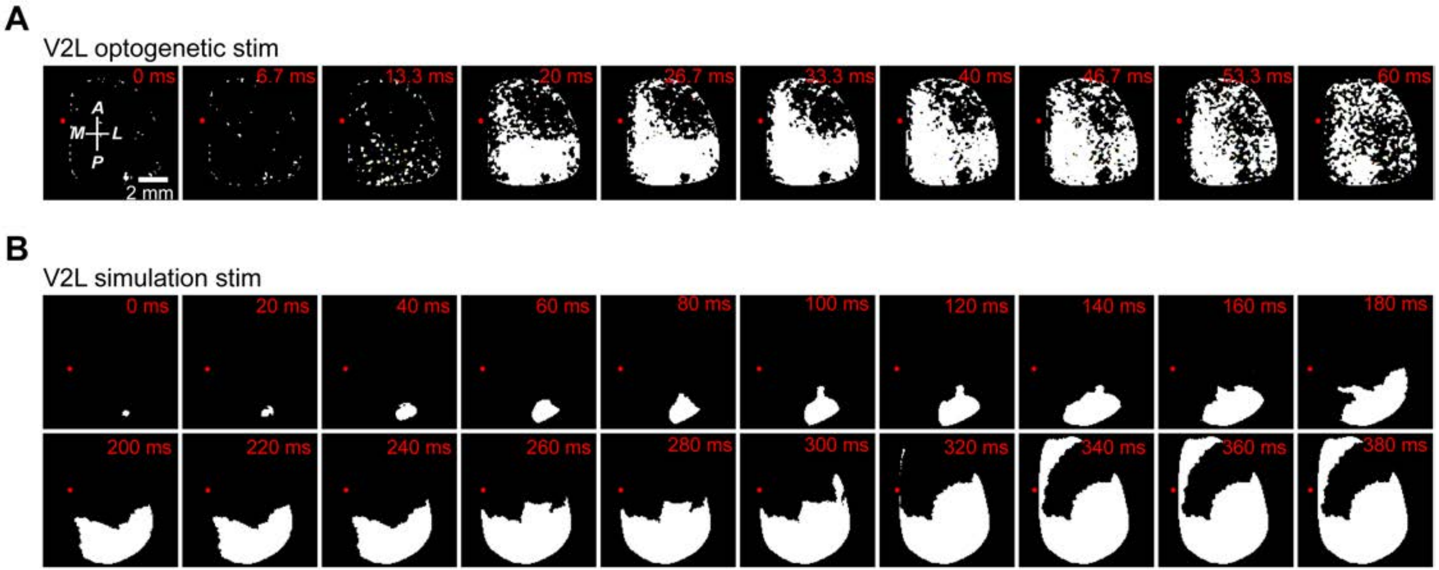

Supplementary Figure 12. Spatiotemporal pattern of activated cortical regions after optogenetic (**A**) and simulated (**B**) stimulation of V2L. A region is defined as activated when its activity surpasses 20 percent of its peak activity after stimulation occurred. (**A**) Schematics of the two axes of polarity AP and ML of V2L in (**A**) and evolution of activated cortical region (in white pixel) with time in (**A**) and (**B**).

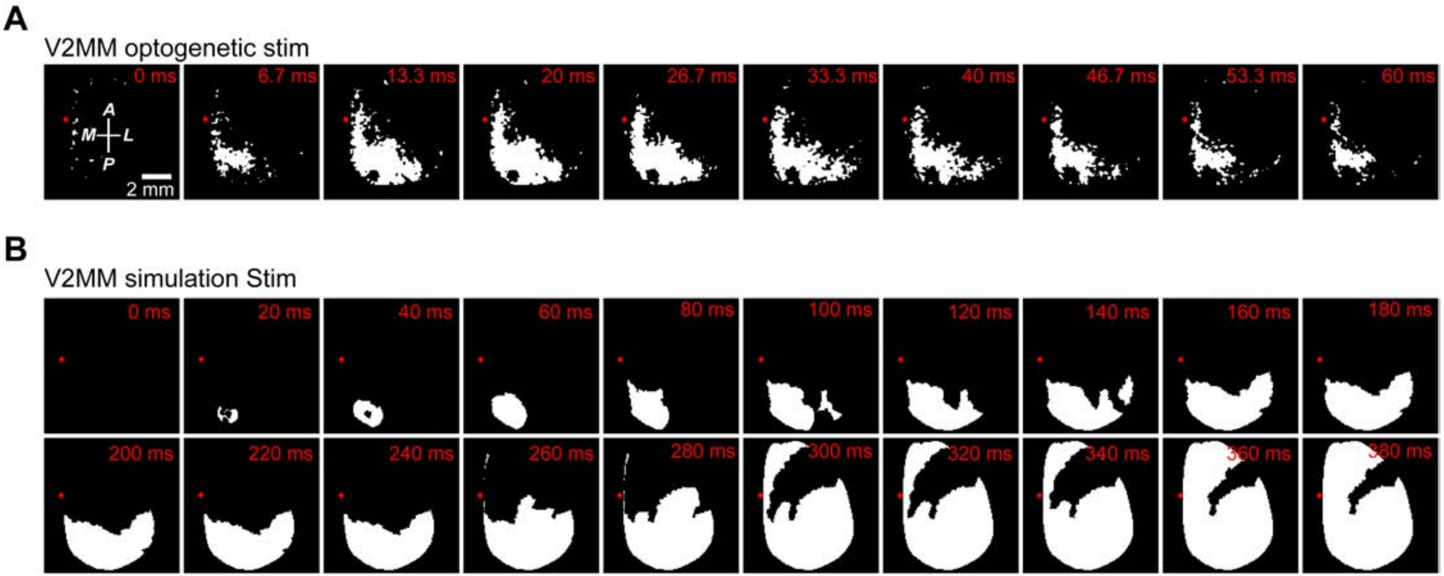

Supplementary Figure 13. Spatiotemporal pattern of activated cortical regions after optogenetic (**A**) and simulated (**B**) stimulation of V2MM. A region is defined as activated when its activity surpasses 20 percent of its peak activity after stimulation occurred. (**A**) Schematics of the two axes of polarity AP and ML of V2MM in (**A**) and evolution of activated cortical region (in white pixel) with time in (**A**) and (**B**).

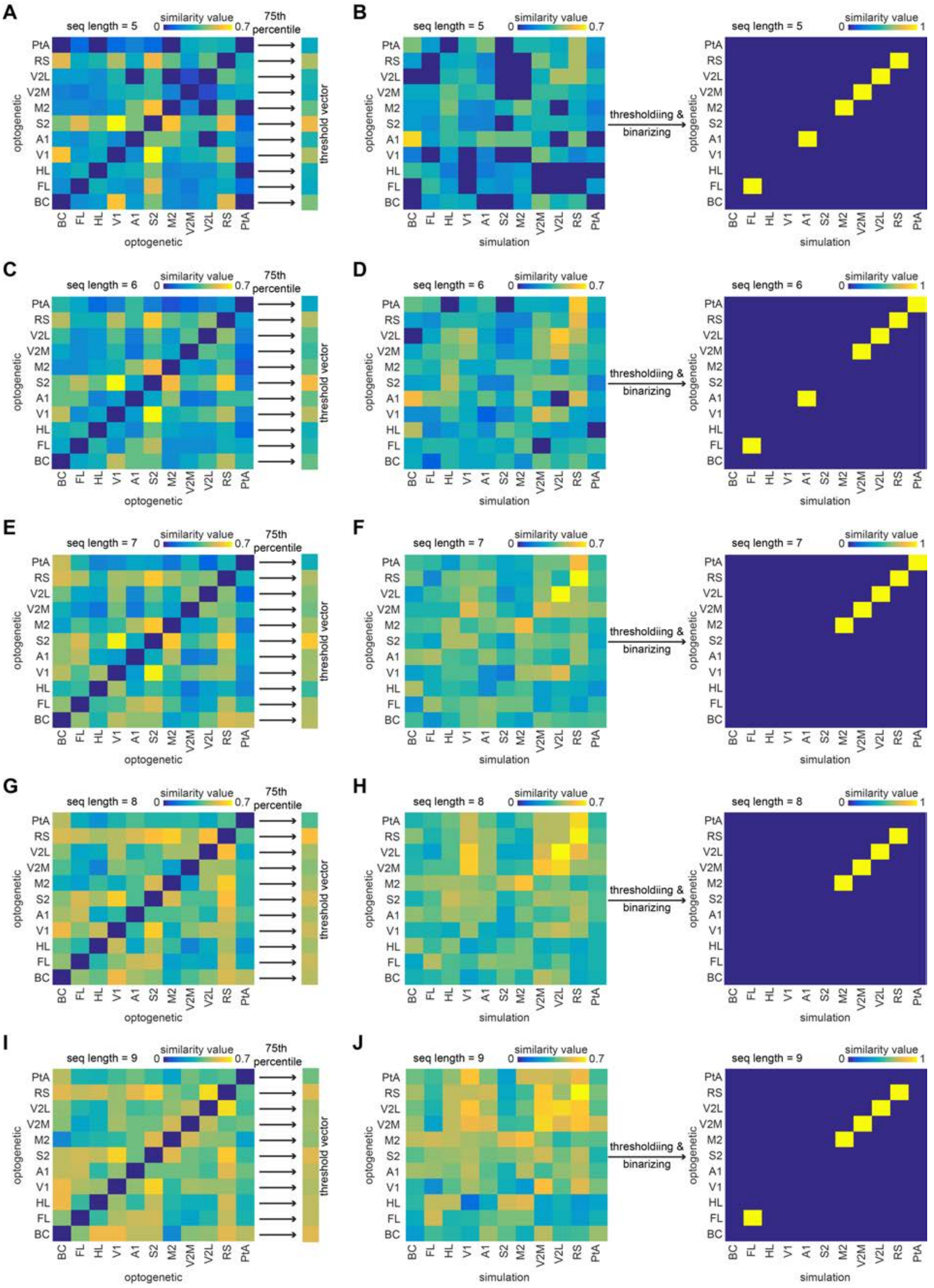

Supplementary Figure 14. The role of sequence length in similarity values between the empirical and the simulated stimulus-induced spatiotemporal pattern of activity in the neocortex. Panel **A**: The similarity matrix consisting of the similarity values between all the pairs of the temporally-ordered sequences of activated regions with length five induced by optogenetic stimulation of neocortical regions. The values on the diagonal of the matrix (self-similarity, which is always 1) were purposefully removed. The 75th percentile of each row gives an expected value for the similarity of the spatiotemporal patterns of activity induced by stimulation of the corresponding and rest of regions. Panel **B**: (Left) The same as panel A but for similarity values between empirical versus simulated spatiotemporal patterns of activity induced by focal stimulation of different neocortical regions. (Right) The similarity matrix between empirical and simulated patterns was thresholded and binarized using the threshold vector generated in Panel A. To highlight how similar the empirical and simulated patterns were, only the values on the diagonal are presented. Panels **C**-**D**, **E**-**F**, **G**-**H,** and **I**-**J** are the same as Panels **A**-**B** but for sequences lengths 5, 6, 7, 8, and 9, respectively.

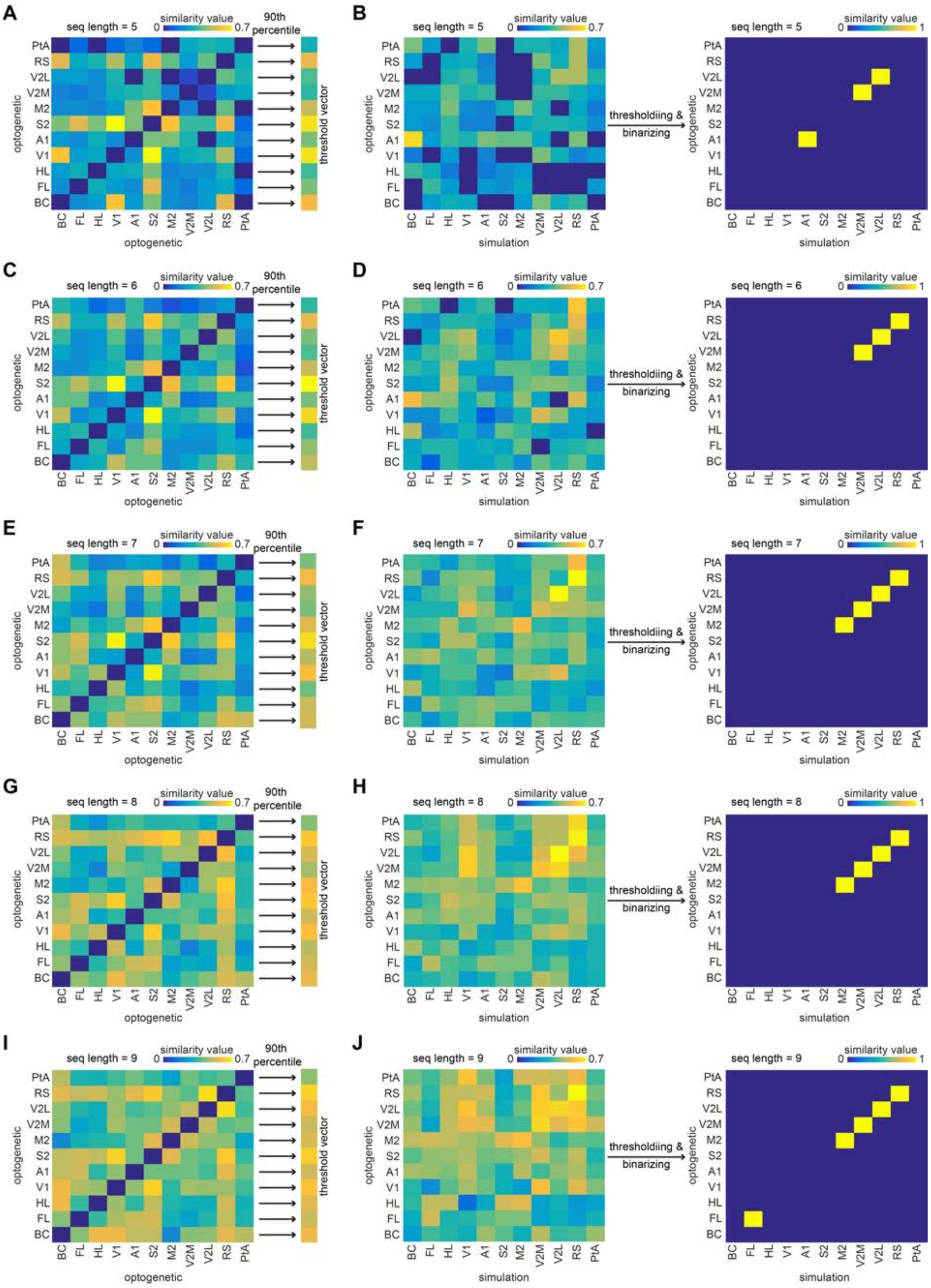

Supplementary Figure 15. The role of sequence length in similarity values between the empirical and the simulated stimulus-induced spatiotemporal pattern of activity in the neocortex. Panel **A**: The similarity matrix consisting of the similarity values between all the pairs of the temporally-ordered sequences of activated regions with length five induced by optogenetic stimulation of neocortical regions. The values on the diagonal of the matrix (self-similarity, which is always 1) were purposefully removed. The 90th percentile of each row gives an expected value for the similarity of the spatiotemporal patterns of activity induced by stimulation of the corresponding and rest of regions. Panel **B**: (Left) The same as panel A but for similarity values between empirical versus simulated spatiotemporal patterns of activity induced by focal stimulation of different neocortical regions. (Right) The similarity matrix between empirical and simulated patterns was thresholded and binarized using the threshold vector generated in Panel A. To highlight how similar the empirical and simulated patterns were, only the values on the diagonal are presented. Panels **C**-**D**, **E**-**F**, **G**-**H,** and **I**-**J** are the same as Panels **A**-**B** but for sequences lengths 5, 6, 7, 8, and 9, respectively.

Supplementary Table 1. Division of brain structures. Number of nodes per area in brackets (left, right).

Isocortex (42 areas)**:**
Frontal pole, cerebral cortex (128, 165)
Primary motor area, M1 (667, 605)
Secondary motor area, M2 (489, 483)
Primary somatosensory area S1, nose (167, 185)
Prim. somatosensory area S1, barrel field (212, 215)
Primary somatosensory area S1, lower limb (89, 85)
Primary somatosensory area S1, mouth (269, 265)
Prim. somatosensory area S1, upper limb (148, 153)
Primary somatosensory area S1, trunk (58, 53)
Prim. somatosensory area S1, unassigned (88, 100)
Supplemental somatosensory area, S2 (271, 223)
Gustatory areas (144, 141)
Visceral area, Visc (94, 120)
Dorsal auditory area (147, 173)
Primary auditory area, A1 (125, 113)
Ventral auditory area (112, 138)
Anterolateral visual area, V2 (58, 63)
Anteromedial visual area, V2 (52, 59)
Lateral visual area, V2 (56, 61)
Primary visual area, V1 (415, 404)
Posterolateral visual area (126, 117)
Posteromedial visual area, V2 (92, 78)
Laterointermediate area, LI (47, 46)
Postrhinal area (130, 134)
Anterior cingulate area, dorsal part (224, 246)
Anterior cingulate area, ventral part (147, 138)
Prelimbic area (105, 98)
Infralimbic area (106, 117)
Orbital area, lateral part (108, 96)
Orbital area, medial part (90, 88)
Orbital area, ventrolateral part (125, 100)
Agranular insular area, dorsal part (169, 173)
Agranular insular area, posterior part (151, 147)
Agranular insular area, ventral part (140, 138)
Retrosplenial area, RSC, lateral agranular (132, 162)
Retrosplenial area, RSC, dorsal part (329, 251)
Retrosplenial area, RSC, ventral part (410, 377)
Anterior area (47, 32)
Rostrolateral visual area (69, 71)
Temporal association areas (145, 148)
Perirhinal area (188, 228)
Ectorhinal area, EC (151, 163)

Olfactory areas (11 areas)**:**
Main olfactory bulb (1, 1)
Accessory olfactory bulb (1, 1)
Anterior olfactory nucleus (1, 1)
Taenia tecta (1, 1)
Dorsal peduncular area (1, 1)
Piriform area (1, 1)
Nucleus of the lateral olfactory tract (1, 1)
Cortical amygdalar area, anterior part (1, 1)
Cortical amygdalar area, posterior part (1, 1)
Piriform-amygdalar area (1, 1)
Postpiriform transition area (1, 1)

Hippocampal Formation (10 areas)**:**
Field CA1
Field CA2
Field CA3
Dentate gyrus
Entorhinal area, lateral part
Entorhinal area, medial part, dorsal zone
Parasubiculum
Postsubiculum
Presubiculum
Subiculum

Cortical Subplate (7 areas)**:**
Claustrum
Endopiriform nucleus, dorsal part
Endopiriform nucleus, ventral part
Lateral amygdalar nucleus
Basolateral amygdalar nucleus
Basomedial amygdalar nucleus
Posterior amygdalar nucleus

Supplementary Table 2. Subcortical brain structures and their division. One network node per area.

**Cerebral Nuclei** (21 areas)**:**	Anteroventral nucleus
Caudoputamen	Anteromedial nucleus
Nucleus accumbens	Anterodorsal nucleus
Fundus of striatum	Interanteromedial nucleus
Olfactory tubercle	Interanterodorsal nucleus
Lateral septal nucleus, caudal (caudodorsal) part	Lateral dorsal nucleus, LD
Lateral septal nucleus, rostral (rostroventral) part	Intermediodorsal nucleus
Lateral septal nucleus, ventral part	Mediodorsal nucleus
Septofimbrial nucleus	Submedial nucleus
Anterior amygdalar area	Perireunensis nucleus
Bed nucleus of the accessory olfactory tract	Paraventricular nucleus
Central amygdalar nucleus	Parataenial nucleus
Intercalated amygdalar nucleus	Nucleus of reunions
Medial amygdalar nucleus	Rhomboid nucleus
Globus pallidus, external segment	Central medial nucleus
Globus pallidus, internal segment	Paracentral nucleus
Substantia innominata	Central lateral nucleus
Magnocellular nucleus	Parafascicular nucleus
Medial septal nucleus	Reticular nucleus
Diagonal band nucleus	Lateral geniculate group, intergeniculate leaflet, IGL
Triangular nucleus of septum	Lateral geniculate complex, ventral part, vLGN
Bed nuclei of the stria terminalis	Medial habenula
**Thalamus** (38 areas)**:**	**Hypothalamus** (33 areas)**:**
Ventral anterior-lateral complex	Paraventricular nucleus
Ventral medial nucleus	Periventricular nucleus, intermediate part
Ventral posterolateral nucleus, VPL	Arcuate nucleus, Arc
Ventral posteromedial nucleus, VPM	Anterodorsal preoptic nucleus
Ventral posteromedial nucleus, parvicellular part	Anteroventral preoptic nucleus
Subparafascicular nucleus, magnocellular part	Anteroventral periventricular nucleus
Subparafascicular nucleus, parvicellular part	Dorsomedial nucleus
Subparafascicular area	Median preoptic nucleus
Peripeduncular nucleus	Medial preoptic area
Medial geniculate complex, MG	Parastrial nucleus
Lateral geniculate complex, dorsal part, dLGN	Periventricular nucleus, posterior part
Lateral posterior nucleus, LPN	Periventricular nucleus, preoptic part
Posterior complex, Po	Subparaventricular zone, SPVZ
Posterior limiting nucleus	Suprachiasmatic nucleus, SCN
Suprageniculate nucleus	Anterior nucleus, AH

The table is continued on the next two pages.

Subcortical brain structures and their division (continued).

**Hypothalamus** (continued)	Cuneiform nucleus
Lateral mammillary nucleus, LM	Red nucleus
Medial mammillary nucleus	Oculomotor nucleus
Supramammillary nucleus	Edinger-Westphal nucleus
Tuberomammillary nucleus, dorsal part	Ventral tegmental nucleus
Tuberomammillary nucleus, ventral part	Substantia nigra, compact part
Medial preoptic nucleus	Pedunculopontine nucleus
Dorsal premammillary nucleus	Interfascicular nucleus raphe
Ventral premammillary nucleus	Interpeduncular nucleus
Paraventricular nucleus, descending division	Rostral linear nucleus raphe
Ventromedial nucleus	Central linear nucleus raphe
Posterior nucleus	Dorsal nucleus raphe
Lateral area	
Lateral preoptic area	**Pons** (17 areas)
Parasubthalamic nucleus	Nucleus of the lateral lemniscus, NLL
Retrochiasmatic area	Principal sensory nucleus of the trigeminal, PrV
Subthalamic nucleus	Parabrachial nucleus
Tuberal nucleus	Barrington's nucleus
Zona incerta	Pontine central gray
	Pontine gray
**Midbrain** (30 areas)	Pontine reticular nucleus, caudal part
Superior colliculus, sensory related, SuCo	Supratrigeminal nucleus
Inferior colliculus, IC	Tegmental reticular nucleus
Nucleus of the brachium, inferior colliculus, BIC	Motor nucleus of trigeminal
Nucleus sagulum	Superior central nucleus raphe
Parabigeminal nucleus	Locus ceruleus
Trigeminal nucleus, MeV	Laterodorsal tegmental nucleus
Substantia nigra, reticular part	Nucleus incertus
Ventral tegmental area	Pontine reticular nucleus
Midbrain reticular nucleus, retrorubral area	Subceruleus nucleus
Midbrain reticular nucleus	Sublaterodorsal nucleus
Superior colliculus, motor related	
Periaqueductal gray	**Medulla** (32 areas)
Anterior pretectal nucleus	Dorsal cochlear nucleus, DC
Medial pretectal area	Ventral cochlear nucleus, VC
Nucleus of the optic tract, OT	Cuneate nucleus, CN
Nucleus of the posterior commissure	Gracile nucleus, GN
Olivary pretectal nucleus, OPT	Nucleus of the trapezoid body, Tz
Posterior pretectal nucleus, PPT	Nucleus of the solitary tract

Subcortical brain structures and their division (continued).

**Medulla** (continued)	Hypoglossal nucleus
Spinal nucleus of the trigeminal, caudal part, SpVc	Nucleus raphe magnus
Spinal nucl. of the trigeminal, interpolar part, SpVi	Superior vestibular nucleus
Spinal nucleus of the trigeminal, oral part, SpVo	Nucleus x
Abducens nucleus, AN	Spinal vestibular nucleus
Facial motor nucleus	
Nucleus ambiguus, NA	**Cerebellum** (15 areas)
Gigantocellular reticular nucleus	Lingula (I)
Inferior olivary complex	Central lobule
Intermediate reticular nucleus	Culmen
Linear nucleus of the medulla	Folium-tuber vermis (VII)
Lateral reticular nucleus	Pyramus (VIII)
Magnocellular reticular nucleus	Nodulus (X)
Medullary reticular nucleus, dorsal part	Simple lobule
Medullary reticular nucleus, ventral part	Ansiform lobule
Parvicellular reticular nucleus	Paramedian lobule
Paragigantocellular reticular nucleus, dorsal part	Copula pyramidis
Paragigantocellular reticular nucleus, lateral part	Paraflocculus
Nucleus of Roller	Flocculus
Nucleus prepositus	Fastigial nucleus
Lateral vestibular nucleus	Interposed nucleus
Medial vestibular nucleus	Dentate nucleus
